# Effects of Immersive Virtual Reality Simulation on Nursing Students’ Learning Outcomes: A Systematic Review and Meta‐Analysis

**DOI:** 10.1155/jonm/4686851

**Published:** 2026-05-15

**Authors:** Xia Li, Yanling Hu, Hanmei Peng, Shiqi Luo, Chunsong Yang, Biru Luo, Xingli Wan, Yuan Li

**Affiliations:** ^1^ Department of Nursing, West China Second University Hospital, Sichuan University, Chengdu, 610041, China, scu.edu.cn; ^2^ Key Laboratory of Birth Defects and Related Diseases of Women and Children (Sichuan University), Ministry of Education, Chengdu, 610041, China, meb.gov.tr; ^3^ Department of Neonatology, West China Second University Hospital, Sichuan University, Chengdu, 610041, China, scu.edu.cn; ^4^ Department of Pharmacy, West China Second University Hospital, Sichuan University, Chengdu, 610041, China, scu.edu.cn; ^5^ Evidence-Based Pharmacy Center, West China Second University Hospital, Sichuan University, Chengdu, 610041, China, scu.edu.cn

**Keywords:** education, learning outcomes, nursing, students, virtual reality

## Abstract

**Background:**

Traditional nursing education faces significant challenges in bridging theoretical knowledge and clinical practice, with clinical placements increasingly constrained by limited availability and patient safety concerns. Immersive virtual reality (IVR) simulation offers unprecedented sensory immersion and spatial presence as a potential solution. However, existing evidence regarding IVR’s effectiveness in nursing education remains fragmented and inconclusive.

**Objective:**

To evaluate the effectiveness of IVR simulation on nursing students’ learning outcomes using Bloom’s taxonomy.

**Methods:**

This systematic review and meta‐analysis followed PRISMA guidelines and included randomized controlled trials comparing IVR simulation with conventional education in nursing students. MEDLINE, Embase, CINAHL, and CENTRAL were searched from inception to March 25, 2025. Outcomes were classified into cognitive, psychomotor, and affective domains according to Bloom’s taxonomy. Random‐effects models were applied, with subgroup and sensitivity analyses conducted to explore sources of heterogeneity and identify potential effect modifiers. The certainty of evidence was assessed using the Grading of Recommendations, Assessment, Development, and Evaluations (GRADE) framework.

**Results:**

Twenty RCTs involving 2063 nursing students were included. In the cognitive domain, IVR simulation significantly improved knowledge acquisition (SMD = 0.33, 95% CI: 0.04–0.61); numerical trends suggested stronger effects when debriefing was incorporated, and benefits diminished at follow‐up. In the psychomotor domain, IVR simulation did not significantly enhance skill performance (SMD = 0.49, 95% CI: −0.24–1.22), despite a numerical trend favoring the intervention. In the affective domain, IVR simulation significantly improved self‐efficacy (SMD = 0.38, 95% CI: 0.04–0.72) but showed no significant effects on satisfaction or confidence. Adverse effects were generally mild and consisted primarily of visual and vestibular symptoms.

**Conclusion:**

IVR simulation may enhance knowledge acquisition and self‐efficacy among nursing students. The importance of debriefing and the need for reinforcement strategies to sustain knowledge gains were highlighted. IVR’s effects on skills and other affective outcomes remain inconclusive. Further high‐quality RCTs with standardized protocols are required to clarify IVR’s role in nursing education.

## 1. Introduction

Nurses constitute nearly half of the global health workforce, making the quality of nursing education a critical determinant of future healthcare delivery [1]. A fundamental challenge in nursing education lies in effectively bridging the gap between theoretical knowledge and clinical practice, ensuring that students develop the competencies necessary for safe and effective patient care [2, 3]. Traditional nursing education relies predominantly on didactic methods such as lectures and isolated skills training, which emphasize knowledge acquisition but inadequately replicate the dynamic complexities of authentic clinical environments [4]. Consequently, this pedagogical disconnect frequently results in “reality shock” when students transition to clinical practice, where they must integrate multiple competencies simultaneously while making time‐critical decisions under pressure [5]. Moreover, clinical placements, while invaluable, are increasingly constrained by limited availability, patient safety concerns, and variability in learning opportunities, further exacerbating barriers to consistent competency development [6].

Simulation‐based learning (SBL) has emerged as a transformative pedagogical approach that effectively addresses these limitations [7]. By immersing students in controlled learning environments with high‐fidelity manikins or standardized patients, SBL creates a psychologically safe setting for deliberate practice and “safe failure,” fostering contextualized clinical reasoning [8]. Within the spectrum of simulation modalities, virtual reality (VR) simulation has emerged as a particularly promising innovation, gaining substantial traction in nursing education [9]. By creating highly realistic virtual clinical scenarios, VR technology enables learners to observe, explore, and interact through embodied engagement, promoting experiential learning that has been shown to enhance motivation, deepen conceptual understanding, and facilitate long‐term knowledge retention [10, 11].

Immersive virtual reality (IVR), characterized by head‐mounted display technology and stereoscopic 3D visualization, represents the most advanced form of VR simulation, offering unprecedented levels of sensory immersion and spatial presence [12, 13]. Unlike desktop‐based VR or traditional simulation methods, IVR enables users to navigate and manipulate virtual environments through natural head movements, hand gestures, and spatial positioning, creating a first‐person experiential learning paradigm that closely mimics real‐world clinical encounters [14, 15]. Central to IVR’s educational efficacy is its capacity to generate “presence”—the subjective feeling of being physically located within the virtual environment—which triggers authentic cognitive and emotional responses that facilitate meaningful learning [5].

Applications of IVR in nursing education span diverse clinical domains, including emergency care scenarios, medication administration procedures, patient assessment protocols, and interprofessional collaboration exercises [16]. IVR has proven particularly effective for teaching complex procedural skills such as endotracheal suctioning, wound care management, and cardiopulmonary resuscitation, where spatial awareness and psychomotor precision are critical [17, 18]. Additionally, IVR’s ability to simulate rare or high‐risk clinical situations, such as cardiac arrest responses or mass casualty events, provides students with exposure to critical scenarios that would be difficult or impossible to replicate in traditional clinical settings [19, 20]. Compared to conventional simulation modalities, IVR offers distinctive advantages, including unlimited repeatability, standardized learning experiences, elimination of geographical constraints, and long‐term cost‐effectiveness, positioning it as a potentially transformative educational tool [16].

Recent systematic reviews focusing on IVR demonstrate its significant potential in healthcare education, yet the evidence base remains fragmented and inconclusive [5, 13, 21]. Specifically, reviews by Quah et al. [5] and Hsieh et al. [13] reported positive outcomes, suggesting that IVR augments the learning experience and effectively improves knowledge, skills, and problem‐solving abilities. Conversely, Liu et al. [21] found that while students had positive experiences, IVR did not demonstrate statistically significant improvements in learning outcomes compared to conventional teaching methods. These conflicting findings reflect several methodological limitations that compromise the robustness of existing evidence, including the incorporation of heterogeneous populations that mix students and licensed professionals, the combination of randomized controlled trials (RCTs) with quasi‐experimental studies, and insufficient specificity to nursing education, all of which limit the applicability and reliability of current conclusions.

To address these evidence gaps, this systematic review and meta‐analysis aimed to evaluate the specific effects of IVR simulation on nursing students’ learning outcomes. By exclusively including RCTs and focusing solely on nursing student populations, this study provided more robust causal inferences and targeted evidence synthesis. Given the multidimensional nature of nursing competency development, which encompasses knowledge acquisition, psychomotor skill mastery, and professional attitude formation, we employed Bloom’s taxonomy as a comprehensive analytical framework to systematically categorize outcomes across cognitive, psychomotor, and affective domains, thereby enabling a nuanced and comprehensive assessment of IVR’s educational impact on nursing students [22–24].

## 2. Methods

### 2.1. Study Design

The reporting of this systematic review and meta‐analysis followed the PRISMA (Preferred Reporting Items for Systematic Reviews and Meta‐analyses) guidelines (Supporting File 1) [25]. The methodological approach was conducted in accordance with the Cochrane Handbook for Systematic Reviews of Interventions. The protocol was registered with PROSPERO (CRD420251039287).

### 2.2. Search Strategy

Systematic searches were conducted in MEDLINE (via Ovid), Embase (via Ovid), CINAHL (via EBSCOhost), and the Cochrane Central Register of Controlled Trials (CENTRAL, via the Cochrane Library) from database inception to March 25, 2025. A comprehensive search strategy was developed in consultation with a medical librarian, combining Medical Subject Headings (MeSH) with free‐text terms tailored to each database. No restrictions were imposed on language or publication year (see Supporting File 2 for the search strategy). To ensure comprehensive coverage, we manually screened the reference lists of all included studies and relevant reviews. Additionally, trial registries, including the World Health Organization International Clinical Trials Registry Platform (ICTRP) and ClinicalTrials.gov, were searched to identify unpublished or ongoing studies.

### 2.3. Eligibility Criteria

Inclusion criteria were defined as follows: (1) Population: nursing students at any educational level. (2) Intervention: IVR simulation delivered via head‐mounted devices (e.g., headsets or VR glasses). (3) Comparison: any control intervention not involving IVR, such as traditional lectures, textbook learning, video‐based instruction, non‐immersive computer simulations, and manikin‐based training. (4) Outcomes: primary outcomes included knowledge acquisition and practical skill performance; secondary outcomes encompassed satisfaction, confidence, self‐efficacy, and other psychosocial measures. Learning outcomes were categorized according to Bloom’s Taxonomy framework, with knowledge assigned to the cognitive domain, skill performance to the psychomotor domain, and satisfaction, confidence, and self‐efficacy to the affective domain [22–24]. (5) Study design: RCTs of all types (including standard RCTs, pragmatic RCTs, non‐inferiority RCTs, and pilot or feasibility RCTs). Studies were included if they employed genuine randomization, regardless of author terminology.

Exclusion criteria included (1) studies with mixed populations (e.g., nursing students and nursing staff) where data for the student subgroup were not reported separately; (2) studies with inadequate or ambiguous descriptions of the IVR intervention; (3) studies reporting only qualitative data, such as interviews or focus group discussions, without quantitative measures; and (4) conference proceedings, abstracts, book chapters, letters, or editorials.

### 2.4. Study Selection and Data Extraction

Two reviewers independently screened the titles and abstracts of all identified records, followed by full‐text assessment of potentially eligible articles according to the predefined inclusion criteria. For all included studies, data were independently extracted by the same two reviewers using a prepiloted standardized data extraction form. Any discrepancies were resolved through discussion or, when necessary, consultation with a third reviewer until consensus was achieved.

### 2.5. Risk of Bias Assessment and Certainty of Evidence Evaluation

Risk of bias in the included studies was assessed using the revised Cochrane risk‐of‐bias tool for randomized trials (RoB 2.0) to evaluate bias across five domains: randomization process, deviations from intended interventions, missing outcome data, measurement of the outcome, and selection of the reported results [26]. Risk of bias was assessed separately for objective and subjective outcomes. Two reviewers independently conducted the assessment, with disagreements resolved through discussion; when consensus could not be reached, a third reviewer provided the final judgment. The overall quality of evidence was assessed using the Grading of Recommendations Assessment, Development, and Evaluation (GRADE) framework.

### 2.6. Statistical Analysis

A narrative synthesis was conducted for all included studies. For meta‐analyses, random‐effects models were employed to account for anticipated heterogeneity. Given that all outcomes in this review were continuous variables, standardized mean differences (SMDs) calculated as Hedges’ g with 95% confidence intervals (CIs) were computed to accommodate differing measurement scales across studies. The magnitude of the pooled Hedges’ g was interpreted as small (∼0.2), medium (∼0.5), or large (∼0.8) based on conventional benchmarks for effect sizes [27]. Heterogeneity was assessed using *I* ^2^ statistics and visual inspection of forest plots, with values of < 25%, 25%–49%, 50%–74%, and ≥ 75% representing low, moderate, substantial, and high heterogeneity, respectively. Subgroup analyses were conducted for primary outcomes when at least six studies reported data for a given outcome, to explore sources of heterogeneity and identify potential trial‐level effect modifiers [28]. Publication bias was assessed through visual inspection of funnel plots and statistically tested using Egger’s regression test. To assess the robustness of pooled estimates, sensitivity analyses were performed using the leave‐one‐out method. Results were visualized using forest plots. All statistical analyses and graphical representations were carried out using *R* statistical software (Version 4.3.2).

## 3. Results

**FIGURE 1:**
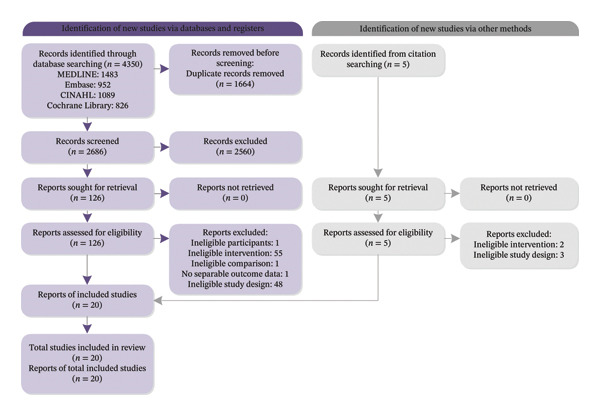
Flow diagram of the study selection process according to PRISMA guidelines.

The PRISMA flowchart for study selection is presented in Figure 1. A total of 4350 records were identified through database searches and five additional records through citation searching. After screening, 20 studies involving 2063 nursing students were included in the review [17–20, 29–44].

### 3.1. Study Characteristics

Geographically, 15 studies were conducted in high‐income countries, one in an upper‐middle‐income country, and four in lower‐middle‐income countries [45]. All 20 included studies were RCTs that employed genuine randomization procedures. Among these, 14 were conventional parallel‐group RCTs, one was a pragmatic RCT, and five employed mixed‐methods or pre–post designs alongside randomization. Notably, three of these studies were originally labeled as “quasi‐experimental” by their authors but employed genuine randomization procedures [35, 40, 43], thus meeting the inclusion criteria for RCTs.

Participants comprised nursing students across all academic levels, with several studies specifically targeting students enrolled in particular courses (e.g., Adult Nursing Practicum, Community Health, and Maternity Nursing). Where reported, female students constituted the majority of participants, ranging from 53% to 100% across studies. Intervention characteristics varied considerably. Seventeen studies reported VR session duration, ranging from 2 to 3 min to 2 h per session. Thirteen studies described single‐session interventions, while seven implemented multiple sessions. All studies incorporated prebriefing, whereas only eight explicitly described postsimulation debriefing procedures. All studies employed head‐mounted display devices for VR delivery. Seventeen studies used advanced VR headsets (including Meta Quest 2, HTC Vive series, Oculus devices, and Windows Mixed Reality systems) with sophisticated tracking capabilities and interactive controllers, while three studies utilized smartphone‐based VR systems (Google Cardboard, stereoscopic lenses with mobile phones, and VR Box systems) that provided more basic immersive functionality. Control groups typically received conventional teaching methods, including traditional lectures, video demonstrations, manikin‐based training, and face‐to‐face instruction. Study outcomes were categorized using Bloom’s taxonomy. Knowledge acquisition and skill performance (cognitive and psychomotor domains) were measured through standardized tests, multiple‐choice questionnaires, practical skill checklists, and structured performance evaluations. Affective outcomes included satisfaction, confidence, self‐efficacy, and communication competency, assessed primarily through self‐reported scales and questionnaires. Detailed study characteristics are summarized in Table 1.

**TABLE 1:** The characteristics of included studies (*N* = 20).

Study (country)	Study design	Setting	Participants/sample size (percentage of Fs)	Study scenarios/learning objectives	Intervention	Comparison	Outcomes (Bloom taxonomy)	Measurement/assessment timepoint
Aydın Doğan & Yazıcı [29] (Turkey: UMIC)	Single‐blind RCT	University—Midwifery Dept.	Final‐year midwifery students *N* = 84 (NR) I/C randomized: 42/42 I/C analyzed: 42/43	Teach week‐by‐week fetal and placental development (Gestation Weeks 6–40)	Fetal Development Application Created by VR Technology (FDAC‐VRT) Facilitation: Technical expert present for support Instruments: Oculus HMD (v1.22.3) with touchpad‐reticle interface Prebriefing: Yes Duration: Average 20 min Session: 1 session Debriefing: NR	Traditional 2‐h theory training (PPT, 54 slides)	Cognitive: knowledge; Adverse effects: NR	Knowledge: Fetal Development Assessment Information Scale (FDAIS) (Baseline, 6 wks post‐int’)

Babaita et al. [30] (Japan: HIC)	RCT (open‐label, parallel 1:1)	Hiroshima University, Japan	3rd‐year undergraduate nursing students (Adult Nursing Practicum) *N* = 62 (100% F) I/C randomized: 31/31 I/C analyzed: 27/30	Teach closed tracheal suction (incl. oral suction) in mechanically ventilated ICU patients	360° VR video showing procedure by certified nurse on high‐fidelity mannequin Facilitation: critical care nurse available Instruments: Meta Quest 2 HMD Prebriefing: Yes Duration: 18 min Session: 1 session Debriefing: NR	Face‐to‐face demonstration of the same procedure by the same nurse	Cognitive: knowledge; Psychomotor: skill performance; Affective: satisfaction, confidence; Adverse Effects: reported	Knowledge: Questions (1‐wk post‐int’); Skills: Checklist (1‐wk post‐int’); Sat/Conf: Scale (1‐wk post‐int’); Safety: Q (post‐int’)

Bani Salameh et al. [31] (Jordan: LMIC)	Pre–post‐test control group design (with randomization)	Al‐Zaytoonah University, Jordan	Nursing students (Community health and pediatric/newborn clinical courses) *N* = 104 (62.7% F) I/C randomized: 52/50 I/C analyzed: 52/50	Teach five procedures related to pediatric and newborn nursing and community nursing clinical courses	VR simulation experiences and traditional teaching (lectures and demonstrations in a laboratory) Facilitation: VR simulation coordinator operated VR procedures and observed progression Instruments: HTC Vive (HMD, headphones, digital BP machine, cameras) Prebriefing: Yes Duration: Max 30 min per procedure Session: 2 sessions Debriefing: Yes	Traditional teaching only (3–4 h lectures and demonstrations)	Psychomotor: skill performance; Affective: satisfaction, self‐confidence; Adverse effects: NR	Performance: Checklists (Baseline, post‐int’); Sat/Conf: LSSCL (Baseline, 1 mo post‐int’);

Berg & Steinsbekk [32] (Norway: HIC)	RCT (non‐inferiority, parallel group)	Norwegian University of Science and Technology	1st‐year medical and nursing students *N* = 289 (78.5% F) I/C randomized: 149/140 I/C analyzed: 149/140	Teach ABCDE approach for systematic clinical observation	Individual self‐practice (20 min) in immersive and interactive VR app (VirSam ABCDE) Facilitation: Minimal Prebriefing: Yes Duration: 20 min Session: 1 session Debriefing: NR	Individual self‐practice (20 min) with traditional equipment (digital BP gauge, oximeter, ear thermometer, clock, ABCDE overview sheet)	Psychomotor: documenting 8 observations in correct ABCDE order; Affective: satisfaction; Adverse effects: NR	Knowledge: Documentation order in practical test (5‐min limit) on manikin (immediately postpractice); Satisfaction: Questionnaire (Likert scale) (immediately postpractice)

Berg & Steinsbekk [33] (Norway: HIC)	RCT (non‐inferiority, parallel group)	Norwegian University of Science and Technology	1st‐year medical and nursing students *N* = 289 (82.0% F) I/C randomized: 146/143 I/C analyzed: 146/143	Teach ABCDE approach for systematic clinical observation	Group self‐practice (groups of 3, 20 min) in multiplayer, immersive, interactive VR app (VirSam ABCDE) Facilitation: Minimal Instruments: Oculus Rift S or Oculus Quest HMD and hand controllers Prebriefing: Yes Duration: 20 min Session: 1 session Debriefing: NR	Group self‐practice (groups of 3, 20 min) with traditional equipment (digital BP gauge, oximeter, ear thermometer, clock, ABCDE overview sheet)	Psychomotor: documenting 8 observations in correct ABCDE order; Affective: satisfaction; Adverse effects: NR	Knowledge: Documentation order in practical test (5‐min limit) on manikin (immediately postpractice); Satisfaction: Questionnaire (Likert scale) (immediately postpractice)

Chao et al. [34] (Taiwan, China: HIC)	RCT	College of Nursing, Taipei Medical University	Nursing students (≥ 20 yrs old, no prior NG tube feeding skill) *N* = 45 (86.7% F) I/C randomized: 22/23 I/C analyzed: 22/23	Teach nasogastric (NG) tube feeding nursing skills	Immersive 3D interactive video program on NG tube feeding using VIVEPAPER technology Facilitation: Research assistant assisted with headset fitting and provided help as needed Instruments: HTC Vive HMD and interactive paper booklet (VIVEPAPER) Prebriefing: Yes Duration: 10–20 min Session: 1 session Debriefing: NR	Traditional demonstration DVD video on NG tube feeding	Cognitive: Knowledge; Psychomotor: Skill performance; Affective: Confidence, Satisfaction; Adverse effects: Reported	Knowledge: NGFQ (15 T/F Qs) (pre, post‐immediate, 1 mo post‐int’); Skills: NG tube feeding exam score (approx. 1 mo post‐int’); Confidence: C‐scale (5 items) (pre, post‐immediate, 1 mo post‐int’); Satisfaction: 10‐item Q (post‐int’)

Chen et al. [35] (Taiwan, China: HIC)	Randomized allocated pretest–post‐test quasi‐experimental design	Mackay Medical College in Taiwan	3rd‐year nursing students *N* = 90 (78.5% F) I/C randomized: 45/45 I/C analyzed: 40/39	Teach health assessment and practice course (incl. basic skills, chest, abdominal, limbs/musculoskeletal, neural, head/neck/throat, breast/lymphoid assessments)	Combination of 3D hologram learning module + classroom lectures over 18 weeks Facilitation: Students were shown how to use the reality system, and a teaching assistant was available for supervision Instruments: Windows Mixed Reality Helmet and VR Remote Control (with Health Assessment® and Patient First Patient Condition VR System® software) Prebriefing: Yes Duration: NR Session: multiple sessions over 18 weeks Debriefing: NR	Classroom lectures only (without 3D holograms) over 18 weeks	Psychomotor: Techniques Performance; Cognitive: Knowledge; Adverse effects: NR	Performance: Health Assessment and Practice Techniques Performance tool (post‐test after 18 wks); Knowledge: Health Assessment and Practice Knowledge Assessment tool (pretest baseline, post‐test after 18 wks)

Chou et al. [36] (Taiwan China: HIC)	Pragmatic RCT	University of Science and Technology, Central Taiwan	2nd‐year nursing students *N* = 84 (81% F) I/C randomized: 42/42 I/C analyzed: 42/42	Teach communication skills (4 tasks: self‐introduction, nurse‐patient relationship establishment, interaction, and medical history collection)	VR Communication Simulation (VRCS) program (simulated hospital scenario, interact with virtual patients via voice commands) Facilitation: Researchers explained and assisted students with mounting the HMD and provided assistance as needed Instruments: HTC Vive Focus 3 VR HMD Prebriefing: Yes Duration: 30 min Session: 4 sessions Debriefing: Yes	Nurse‐patient communication teaching video (30 min)	Psychomotor: Communication ability; Affective: Confidence, Satisfaction, Stress; Adverse effects: NR	Comm Ability: Kalamazoo Essential Element Comm Checklist; Comm Confidence: Comm Confidence Self‐Assessment Scale (VAS); Satisfaction: Learning Satisfaction Q; Stress: Stress Scale for Nursing Students (All measured at T0, T1, T2, T3)

Dragnes Brix et al. [17] (Denmark: HIC)	Convergent mixed‐methods RCT	School of Nursing, VIA University College (Collaboration with Horsens Regional Hospital and Aarhus University)	3rd‐year nursing students *N* = 59 (96.6% F) I/C randomized: 30/29 I/C analyzed: 28/22	Teach BLS skills in hospital setting (algorithm, compressions, ventilation, meds, AED use)	Individual VR simulation (“Basic Life Support in the Hospital”) prior to HF simulation + Standard theoretical and hands‐on BLS training (received by both groups) Facilitation: Researchers assisted with mounting the VR headset and provided assistance as needed Instruments: 6 degrees of freedom VR headset (like Quest 3) Prebriefing: Yes Duration: Approx. 10–15 min Session: 1 session Debriefing: NR	Standard theoretical and hands‐on BLS training only, followed by HF simulation	Affective: Confidence, Self‐efficacy (general and BLS), professional competence; Adverse effects: NR	1. Confidence regarding BLS measured by self‐designed questionnaires 2. Self‐efficacy in general/regarding BLS measured by self‐designed questionnaires 3. Professional competency regarding BLS measured by self‐designed questionnaires /pretest and post‐test

Gray et al. [37] (Australia: HIC)	Pilot RCT (two‐armed parallel)	Charles Darwin University	2nd‐year bachelor of midwifery students *N* = 38 (100% F) I/C randomized: 20/18 I/C analyzed at post‐test: 19/17 I/C analyzed at 1‐month: 9/9	Teach physiological process of the third stage of labor (uterine environment, placental separation, hemostasis)	3D midwifery visualization resource (3DMVR) with audio narration on mobile device using stereoscopic lenses + traditional teaching beforehand Facilitation: Self‐directed learning Instruments: Stereoscopic lenses (3D glasses) attached to students’ personal mobile phones with earpieces for audio Prebriefing: Yes Duration: NR Session: 1 session Debriefing: NR	Traditional teaching only	Cognitive: Knowledge; Affective: Satisfaction; Adverse effects: Reported	Knowledge: 30‐item MCQ (Pretest baseline, immediate post‐test, 1‐month follow‐up); Satisfaction: Student Satisfaction with 3DMVR Scale (17 items) (immediately after 3DMVR viewing)

Jeong & Cha [38] (South Korea: HIC)	Single‐blinded, two‐arm RCT	Maternity nursing simulation class at Wonkwang University	Junior (second‐year) nursing students registered for a maternity nursing simulation course *N* = 62 (93.3% F) I/C randomized: 31/31 I/C analyzed: 30/29	Scenario‐based simulation on normal vaginal delivery care (stages 1–4)	Immersive VR (IVR) simulation training (Oculus Quest 2); students interacted with virtual environment/nurse, answered questions to progress Facilitation: Research assistants provided 1‐on‐1 assistance for VR operation Instruments: Oculus Quest 2 HMDs with remote controllers Prebriefing: Yes Duration: 2 h Session: at least twice Debriefing: Yes	Conventional simulation class using SimMom birthing simulator manikin + roleplay; same learning goals/content.	Cognitive: Knowledge; Affective: Satisfaction, Self‐efficacy, Confidence; Adverse effects: NR	Knowledge: 20‐item scale (Hong, 2015); Satisfaction: 5‐item scale (NLN, 2005); Self‐Efficacy: 18‐item scale (Guimond & Simonelli, 2012); Confidence: 8‐item scale (NLN, 2005) /pretest and post‐test

Kim et al. [20] (Republic of Korea: HIC)	Single‐blind RCT	A university nursing department in Gyeonggi‐do, Republic of Korea	Nursing students (aged over 20 years) *N* = 44 (86.4% F) I/C randomized: 23/21 I/C analyzed: 23/21	Teach CPR skills via 3 scenarios (heart attack, drowning, traffic accident), covering assessment, activation, airway, CPR, defibrillation.	VR CPR training (“CPR HEART” equip.): (1) View VR CPR video (5 min), (2) VR device intro/safety (5 min), (3) Select scenario and VR CPR practice (10 min), (4) Observe other student VR CPR (10 min). Total 30 min Facilitation: Research assistants present in lab, provided guidance on VR use/operation/precautions Instruments: VIVE PRO headset, VIVE tracker, chest compression input device (CPR mannequin), high‐performance PC (Intel i7, GTX1070+) Prebriefing: Yes Duration: 30 min Session: 1 session Debriefing: NR	Traditional BLS training (certified instructor‐led: CPR lectures, demo, hands‐on CPR training), approx. 4 h	Cognitive: Knowledge; Affective: Self‐efficacy, Confidence; Psychomotor: Skills; Adverse effects: NR	Knowledge: 10 MCQ (KACPR) (pre/post); Self‐efficacy: 10‐item tool (pre/post); Confidence: 17‐item tool (KABONE modified) (pre/post); Skills: Resusci Junior QCPR (Score, Depth, Rate, Relaxation, Accuracy) (post)

Lee et al. [40] (Hongkong, China: HIC)	Single‐blinded, two‐arm RCT	The University of Hong Kong	Undergraduate nursing students (“Nursing of Adults” course, prelicensure) *N* = 151 (64.2% F) I/C randomized: 75/76 I/C analyzed: 75/76	Teach Blood Transfusion (BT) nursing practice (theory: blood formation, BT reactions; practical skills: BT procedure, problem‐solving scenarios)	Usual 2‐h online lecture (Zoom) + VR simulation videos (Animated 3D: blood formation, reactions—13:24 total; 360° VR: BT procedure, 3 problem‐solving scenarios) watched via Google Cardboard Facilitation: Self‐directed learning Instruments: Handheld smartphone VR headset (Google Cardboard Viewer) used with students’ personal smartphones Prebriefing: Yes Duration: Animated VR: 13 min 24 s; 360° VR: Not specified for entire session (included three 2‐min case videos) Session: Self‐paced multiple sessions within 1 week Debriefing: NR	Usual 2‐h online lecture (Zoom) only	Cognitive: Knowledge; Affective: Satisfaction, Confidence, Self‐efficacy; Adverse effects: NR	Knowledge: RBTKQ‐O (34 items); Satisfaction/Confidence: SSSC scale; Self‐efficacy: GSES/All measured pretest and post‐test (1‐wk after Zoom lecture)

Lee et al. [40] (Republic of Korea: HIC)	Quasi‐experimental study (pre–post design) with randomized allocation	Simulation center of a nursing school located in D district of Korea	4th‐year undergraduate nursing students *N* = 68 (85.3% F) I/C randomized: 32/36 I/C analyzed: 32/36	Teach schizophrenia care via 5 situation‐based learning scenarios (risk of violence, relationship delusion, hallucinations, suicide risk, refusal of medication)	VR simulation program using 360° videos. After each video, students completed tasks (MCQs, attempted conversations with virtual patients) Facilitation: NR Instruments: HMD (Oculus Go by Facebook Inc.) with a hand‐held controller Prebriefing: Yes Duration: Average 34.6 min Session: 1 session Debriefing: NR	Conventional simulation using text scenario‐based role play in groups of 4–5 students	Psychomotor: Mental health nursing performance; Affective: Communication competency; Adverse effects: NR	Mental health nursing performance measured by a 24‐item instrument with five subscales (post‐test); Communication competency measured by a 9‐item tool (pretest and post‐test)

Liao et al. [19] (China: LMIC)	RCT (Two‐arm, randomized, controlled waiting list study)	Tertiary nursing program in Sichuan, China	2nd‐year nursing students registered for a disaster nursing course *N* = 106 (72.3% F) I/C randomized: 53/53 I/C analyzed: 49/52	Teach disaster nursing via 12 scenarios (earthquake, fire, triage, wound dressing, fixation, hemostasis, debridement, CPR, tracheal intubation, transportation, decontamination, psychological care)	VR training scenarios using HTC Vive headsets + Usual disaster nursing course. Each scenario had 3 models: instructing, training, testing Facilitation: Participants had 1 week post‐lecture to access VR lab under supervision; unlimited attempts allowed Instruments: HMDs (HTC Vive) with scenarios developed using Unity 3D engine Prebriefing: Yes Duration: 10–25 min per scenario Session: 12 scenarios, multiple attempts allowed Debriefing: Yes	Usual disaster nursing course only (24 lectures, 4 skills lab manikin sessions)	Cognitive: Knowledge (Disaster Preparedness); Psychomotor: Performance; Affective: Confidence; Adverse Effects: Reported	1. Disaster preparedness measured by Disaster Preparedness Questionnaire (DPQ) 2. Performance measured by five blinded examiners during simulated disaster incident 3. Confidence measured by self‐developed assessment cards /pretest and post‐test

Lo et al. [41] (Taiwan, China: HIC)	RCT (Randomized controlled trial with pretest and post‐test design)	Long‐term care departments of two universities in central Taiwan	University students from Departments of Long‐Term Care *N* = 107 (80.4% F) I/C randomized: 54/53 I/C analyzed: 54/53	Teach nasogastric (NG) tube feeding procedure	IVR situational learning of NG tube feeding procedure (simulated real‐world scenarios, task completion via correct decisions/steps, self‐paced) Facilitation: Researcher observed/protected participants and explained VR operation as needed. Instruments: HMDs (HTC VIVE Focus Plus) with IVR application Prebriefing: Yes Duration: Average 34.6 min Session: 1 session Debriefing: NR	A conventional 15‐min 2D video on nasogastric tube feeding procedures	Cognitive: Knowledge; Affective: Learning motivation, Cognitive load, Satisfaction; Adverse effects: Monitored	Knowledge: 10 MCQ (pre/post‐test); Learning Motivation: Modified MSLQ (post‐test); Cognitive Load: 8‐item Q (post‐test); Learning Satisfaction: 7‐item Q (post‐test)

Mayor Silva et al. [42] (Spain: HIC)	RCT (Randomized and controlled clinical trial with pretest and post‐test)	Faculty of Nursing, Complutense University of Madrid, Spain	1st‐year students from the Faculty of Nursing *N* = 105 (84% F) I/C randomized: 52/53 I/C analyzed: 50/50	Develop communication and interpersonal relationship skills	VR simulation focused on communication skills. Facilitation: A technical expert was present for contingencies prevention and management Instruments: VR goggles and headphones connected to computers in a specially conditioned hall Prebriefing: Yes Duration: NA Session: 1 session Debriefing: Yes	Case‐based traditional workshop (students viewed theory content, completed written case, interactive Q&A forum, instructor feedback on correct answers)	Affective: Communication and interpersonal relationship skills; Adverse effects: NR	Nursing Skills Assessments Scale (ECOEnf), Skill Unit UC6 (Communication and interpersonal relationships)/pretest and post‐test

Plotzky et al. [18] (Germany: HIC)	Mixed‐methods randomized parallel study	Nine nursing and anesthetics technologist schools	2nd‐ and 3rd‐year nursing and anesthetics technologist students *N* = 131 (74% F) VRhigh/VRlow/Video Randomized: 47/41/43 VRhigh/VRlow/Video Analyzed: 47/41/43	Teach Endotracheal Suctioning (ETS) procedure (14 steps, aseptic non‐touch technique)	GroupVRlow: VR simulation with controller input. GroupVRhigh: VR simulation with hand‐tracking + Supporting video clips. Both actively performed ETS in VR following audio/visual guidance Facilitation: Two researchers provided standardized VR intro and helped with predetermined cues if participants got stuck Instruments: Oculus Quest 2 headsets (VRlow: controllers; VRhigh: hand‐tracking) Prebriefing: Yes Duration: 20 min Session: 1 session Debriefing: Yes	A video tutorial showing ETS being performed on a manikin by an expert, with the same explanations, audio, visual hints, and sequence of steps as in the VR simulations	Cognitive: knowledge acquisition and retention; Psychomotor: skill performance; Affective: learner satisfaction; Adverse effects: reported	Knowledge: Self‐developed test (pretest and post‐test); Skills: OSCE (post‐test); Satisfaction: ABC‐SAT (Cognitive subscale) (post‐test)

Rasouli [43] (Iran: LMIC)	Quasi‐experimental study (pretest–post‐test design) with random assignment	Razi Medical School in Kermanshah	4th‐year nursing students enrolled in anatomy courses *N* = 62 (53.2% F) I/C randomized: 31/31 I/C analyzed: 31/31	Teach anatomy over 7 sessions (Topics: General body, Heart anatomy/physiology x2, Respiratory physiology x2, Brain anatomy x2)	VR‐Based Teaching (VRBT): Used VR apps (Anatomy learning‐3D, Living heart VR, Respiratory system anatomy Pro, Brain anatomy Pro VR) on mobile phones with VR Box 105 headsets. Approx. 7–8 min VR per student/session + in‐person classroom tasks (exercises) Facilitation: Professor introduced lesson content. Students operated VR apps using remote control Instruments: Mobile phones + VR Box 105 headsets + wired headphones + remote control Prebriefing: Yes Duration: Approx. 7–8 min of VR scenarios per student per session Session: 7 sessions Debriefing: NR	Traditional Learning (TL): In‐person teaching using PowerPoint/videos + Moodle Cloud resources + collaborative group tasks/exercises + teacher feedback	Cognitive: Learning performance (knowledge)	Knowledge: Academic Achievement Test (pre/post‐test—Wk 8)

Zahran et al. [44] (Palestine: LMIC)	RCT (pre–post‐test control group design)	Arab American University in Palestine	3rd‐year nursing students *N* = 190 (58.9% F) I/C randomized: 95/95 I/C analyzed: 95/95	Teach airway management scenario based on AHA guidelines	VR airway management training sessions. VR experience included written instructions and audio alarms for errors, requiring restart. Facilitation: VR instructor managed lab/equipment and trained students on VR use; researchers provided intervention details Instruments: HMDs with handheld controllers (sticks) Prebriefing: Yes Duration: Approx. 15–20 min per session Session: 8‐9 sessions Debriefing: Yes	Traditional learning: 2 sessions (160–180 min each) over 2 days. Day 1: Theory (lectures, videos, discussion). Day 2: Application (demo, re‐demo on manikins, group work)	Psychomotor: Skill performance; Affective: Self‐efficacy, Emotional intelligence; Adverse Effects: NR	1. Performance: AHA Airway Management Skills Checklist/Post‐test 2. Self‐efficacy: New General Self‐Efficacy Scale (NGSE)/pre and post‐test 3. Emotional Intelligence: Wong Law Emotional Intelligence Scale (WLEIS)/pre and post‐test

*Note:* F, female.

Abbreviations: BLS = basic life support, CPR = cardiopulmonary resuscitation, HIC = high‐income country, HMD = head‐mounted display, ICU = intensive care unit, LMIC = lower‐middle‐income country, NR = not reported, RCT = randomized controlled trial, UMIC = upper‐middle‐income country, VR = virtual reality.

### 3.2. Risk of Bias of Included Studies and Certainty of Evidence

Risk of bias was assessed using RoB 2.0, with separate evaluations for objective outcomes (knowledge acquisition and skill performance) and subjective outcomes (affective measures). For objective outcomes (18 studies), five studies had low risk of bias, 10 raised some concerns, and three had high risk. Two studies showed high risk for the randomization process, one for missing outcome data, and seven raised concerns for outcome measurement. All studies had low risk for selective reporting. For subjective outcomes (17 studies): all studies had high overall risk of bias, with 16 studies showing a high risk for outcome measurement due to reliance on self‐reported measures. Detailed results are presented in Supporting Figure 1 and 2.

**FIGURE 2:**
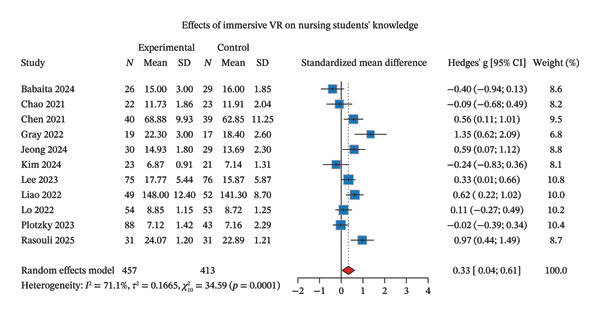
Effectiveness of IVR simulation on knowledge acquisition.

Based on the GRADE framework, the certainty of evidence for knowledge acquisition outcomes was rated as low; skill performance outcomes were rated as very low. Detailed GRADE assessments are provided in Supporting Table 1.

### 3.3. Intervention Effects

#### 3.3.1. Primary Outcomes: Knowledge Acquisition

Within the cognitive domain, knowledge acquisition was the sole outcome assessed. Eleven studies evaluated knowledge acquisition as an outcome measure [18–20, 30, 34, 35, 37–39, 41, 43]. The random‐effects meta‐analysis indicated that IVR significantly improved nursing students’ knowledge acquisition, yielding a small‐to‐medium effect size (Hedges’ *g* = 0.33, 95% CI: 0.04–0.61) according to Cohen’s conventional criteria. However, substantial heterogeneity was observed across studies (*I* ^2^ = 71.1%) (Figure 2). Sensitivity analyses using the leave‐one‐out method demonstrated that results showed some sensitivity to individual studies, as CIs crossed the null line when certain studies were excluded (Supporting Figure 3). The funnel plot appeared symmetrical, and Egger’s test indicated no significant small‐study effects (*p* = 0.611) (Supporting Figure 4).

Subgroup analyses were performed based on the presence of debriefing and the timing of follow‐up assessment to explore sources of heterogeneity and identify potential trial‐level effect modifiers. When stratified by debriefing presence, interventions incorporating debriefing yielded significant improvements in knowledge acquisition (Hedges’ *g* = 0.36, 95% CI: 0.06–0.65), while those without debriefing showed no significant effect (Hedges’ *g* = 0.31, 95% CI: −0.15–0.77). Nevertheless, the test for subgroup differences was not statistically significant (*p* = 0.85). Notably, heterogeneity was reduced to moderate levels in the debriefing subgroup (*I* ^2^ = 54.8%, a reduction of 16.3% points from the overall estimate) but remained substantial in the no‐debriefing subgroup (*I* ^2^ = 78.3%) (Supporting Figure 5). When stratified by follow‐up timing, the immediate post‐intervention assessment showed a significant positive effect (Hedges’ *g* = 0.39, 95% CI: 0.03–0.74), whereas the later follow‐up assessment demonstrated no significant benefit (Hedges’ *g* = 0.19, 95% CI: −0.35–0.72). Similarly, the test for subgroup differences was not statistically significant (*p* = 0.55). However, substantial heterogeneity persisted in both the immediate (*I* ^2^ = 73.6%) and later follow‐up subgroups (*I* ^2^ = 74.4%) (Supporting Figure 6).

#### 3.3.2. Primary Outcomes: Skill Performance

**FIGURE 3:**
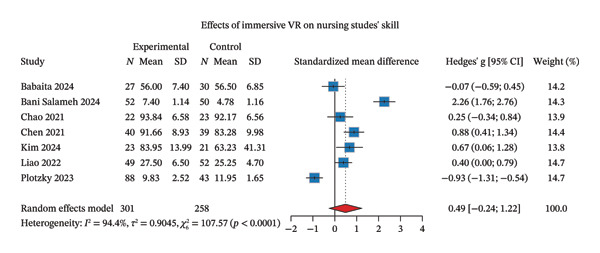
Effectiveness of IVR simulation on skill performance.

Within the psychomotor domain, skill performance was the sole outcome assessed. Seven studies evaluated skill performance as an outcome measure [18–20, 30, 31, 34, 35]. The random‐effects meta‐analysis revealed that IVR did not significantly improve nursing students’ skill performance. The pooled point estimate corresponded to a nominal medium effect size (Hedges’ *g* = 0.49), but the 95% CI was wide and included both no effect and a large effect (95% CI: −0.24–1.22). Very high heterogeneity was observed across studies (*I* ^2^ = 94.4%) (Figure 3). Sensitivity analyses demonstrated that results remained consistent with the original findings, as CIs consistently crossed the null line when individual studies were excluded (Supporting Figure 7). The funnel plot appeared symmetrical, and Egger’s test indicated no significant small‐study effects (*p* = 0.352) (Supporting Figure 8).

When stratified by debriefing presence, interventions without debriefing demonstrated marginally significant improvements in skill performance (Hedges’ *g* = 0.44, 95% CI: 0.00–0.87), while those incorporating debriefing showed a larger but nonsignificant effect (Hedges’ *g* = 0.57, 95% CI: −1.24–2.38). However, the test for subgroup differences was not statistically significant (*p* = 0.89). Notably, heterogeneity was substantially reduced in the no‐debriefing subgroup (*I* ^2^ = 62.8%, a reduction of 31.6% points from the overall estimate) but remained very high in the debriefing subgroup (*I* ^2^ = 98%) (Supporting Figure 9). When stratified by follow‐up timing, the immediate post‐intervention assessment showed a larger but nonsignificant effect (Hedges’ *g* = 0.59, 95% CI: −0.69–1.88) compared to the later follow‐up assessment (Hedges’ *g* = 0.37, 95% CI: −0.20–0.93). The test for subgroup differences was not statistically significant (*p* = 0.75). However, very high heterogeneity persisted in the immediate follow‐up subgroup (*I* ^2^ = 97%), while heterogeneity was reduced to substantial levels in the later follow‐up subgroup (*I* ^2^ = 73.2%, a reduction of 21.2% points) (Supporting Figure 10).

#### 3.3.3. Secondary Outcomes: Affective Domain

Within the affective domain, outcomes assessed included satisfaction, confidence, and self‐efficacy, along with several other measures reported by a very limited number of studies.

Nine studies evaluated satisfaction [18, 30–34, 38, 39, 41], and the random‐effects meta‐analysis indicated that IVR did not significantly enhance nursing students’ satisfaction compared with conventional educational methods (Hedges’ *g* = 0.88, 95% CI: −0.18–1.94), though very high heterogeneity was observed across studies (*I* ^2^ = 95.8%) (Supporting Figure 11). Similarly, eight studies that examined confidence [17, 19, 20, 30, 31, 34, 38, 39] revealed no significant difference between IVR and conventional education (Hedges’ *g* = 0.50, 95% CI: −0.16–1.17), with very high heterogeneity persisting across studies (*I* ^2^ = 91.9%) (Supporting Figure 12). In contrast, five studies assessing self‐efficacy [17, 20, 38, 39, 44] demonstrated that IVR significantly improved nursing students’ self‐efficacy compared with conventional education (Hedges’ *g* = 0.38, 95% CI: 0.04–0.72), with substantial heterogeneity observed across studies (*I* ^2^ = 73.3%) (Supporting Figure 13). Sensitivity analyses for these affective outcomes revealed that the results for satisfaction and confidence remained consistent with the original findings, with CIs consistently crossing the null line when individual studies were excluded, while self‐efficacy was more susceptible to influence from specific studies, with certain individual study exclusions affecting statistical significance (Supporting Figures 14–16).

Additional affective outcomes were assessed by one or two studies but could not be meta‐analyzed due to insufficient data. For communication competency, two studies yielded contrasting results: Lee et al. [40] found no significant differences between IVR and conventional education (Hedges’ *g* = −0.06, 95% CI: −0.54 to 0.42) [40], while Mayor Silva et al. (2023) demonstrated a large significant effect favoring IVR (Hedges’ *g* = 1.25, 95% CI: 0.82–1.68) [42]. For emotional intelligence, one study using the Wong Law Emotional Intelligence Scale found that although both groups improved significantly post‐intervention, the IVR group demonstrated greater gains, with significantly higher post‐test scores compared to controls (*M* = 5.48 vs. *M* = 4.19; *p* < 0.001) [44]. For clinical practice stress, one study reported that although the IVR group had higher baseline stress than controls, this group achieved significantly greater stress reduction at all follow‐up points post‐intervention [36]. Finally, regarding cognitive load, one study found that IVR did not significantly increase cognitive load compared with conventional methods, with numerically higher but nonsignificant post‐intervention levels in the IVR group (*M* = 3.47, SD = 0.74) compared to the 2D video control group (*M* = 3.15, SD = 0.66; *p* = 0.21) [41].

### 3.4. Adverse Effects

Seven studies reported adverse effects associated with IVR interventions. One study documented no adverse events [41], while the remaining six studies reported various side effects [18, 19, 30, 34, 37, 43], predominantly related to visual and vestibular symptoms. The most commonly reported adverse effects included dizziness, nausea, eye fatigue, and blurred vision, with additional symptoms encompassing headache, neck pain, facial discomfort, unsteadiness, and sensations resembling intoxication.

## 4. Discussion

This systematic review and meta‐analysis of 20 RCTs involving 2063 nursing students provides important insights into the differential effectiveness of IVR across learning domains in nursing education. Our findings demonstrate that IVR significantly enhances knowledge acquisition in the cognitive domain, while showing no significant improvement in skill performance within the psychomotor domain. These contrasting results reveal that IVR’s educational impact is domain‐specific rather than universally beneficial, providing crucial guidance for strategic implementation in nursing curricula.

The significant positive effect of IVR on nursing students’ knowledge aligns with established learning theories emphasizing experiential and contextual learning environments [46, 47]. IVR creates engaging three‐dimensional spaces that promote deeper cognitive processing through multisensory integration and spatial presence, facilitating the construction of robust mental models through visualization of complex anatomical structures and realistic clinical scenarios. This finding is consistent with Hsieh et al.’s meta‐analysis, though our exclusive focus on RCTs provides stronger causal evidence [13]. Our subgroup analyses revealed important trends suggesting that debriefing presence may enhance effectiveness, with interventions incorporating structured debriefing sessions showing numerically stronger knowledge gains and reduced heterogeneity. The consistency of the numerical trend with theoretical expectations and the reduction in heterogeneity provide indirect support for this interpretation. Although subgroup differences did not reach statistical significance, this pattern aligns with SBL best practices [48] and suggests that debriefing remains a critical component worthy of systematic inclusion [49–51]. Subgroup analysis by assessment timing revealed that knowledge benefits were primarily observed immediately post‐intervention, with effects attenuating at later follow‐up assessments, highlighting a critical implementation insight: IVR‐based knowledge gains require reinforcement strategies for sustained retention [52, 53].

The lack of significant improvement in psychomotor skill performance, despite theoretical advantages of immersive simulation, provides important insights into current IVR technology limitations and implementation challenges. This finding differs from some previous reviews [5, 13, 21] but reflects the rigorous methodological standards of our RCT‐only approach, which provides more conservative but reliable estimates of effect. Several factors may explain this unexpected result. First, psychomotor skill acquisition in nursing requires precise tactile feedback, fine motor control, and haptic sensations that current IVR technology may not adequately replicate. While IVR excels at providing visual and auditory stimuli, the absence of realistic force feedback and tactile cues may limit its effectiveness for procedures requiring manual dexterity, such as intravenous catheter insertion or wound care management. Second, the duration and intensity of VR training sessions may have been insufficient to achieve mastery of complex psychomotor skills, which typically require extensive deliberate practice. Third, the assessment of skill performance in the included studies varied considerably, ranging from structured checklists to expert evaluations, potentially introducing measurement heterogeneity that obscured true effects. However, the numerical trend favoring IVR (effect size = 0.49) suggests potential benefits that may become significant as technology advances and implementation strategies improve.

The substantial heterogeneity observed across skill performance studies reflects varied IVR effectiveness across different procedural contexts and implementation approaches, though the current evidence base remains insufficient to identify specific factors driving these differences. Subgroup analyses revealed that interventions with debriefing showed numerically larger effects than those without, and immediate post‐intervention assessments demonstrated larger effects than later follow‐up; however, neither subgroup comparison reached statistical significance. This pattern mirrors knowledge acquisition findings, suggesting that debriefing and assessment timing may be important factors across learning domains. These findings suggest that IVR currently serves best as a preparatory or supporting tool rather than a replacement for hands‐on clinical training, particularly for skills requiring sophisticated tactile proficiency, though this may change as haptic technologies advance. For current implementation, educators should prioritize incorporating debriefing sessions and reinforcement strategies aligned with forgetting curve principles to optimize learning outcomes within existing technological constraints.

The analysis of affective outcomes revealed theoretically important patterns with significant implications for IVR implementation in nursing education. Self‐efficacy emerged as the only affective outcome showing significant improvement, while confidence showed no statistically significant difference despite the IVR group demonstrating a numerical tendency toward higher confidence. Self‐efficacy refers to generalized beliefs about one’s capacity to acquire and master skills across different contexts, whereas confidence reflects context‐specific beliefs in performing particular tasks. This distinction underscores IVR’s potential to strengthen learners’ broader sense of professional competence rather than immediate task‐specific confidence. For satisfaction, despite a large effect size favoring IVR, the wide CI suggests considerable variability in student responses, with some experiencing enhanced satisfaction while others may find the technology initially challenging or overwhelming.

The very high heterogeneity observed across satisfaction and confidence studies indicates that affective responses to IVR vary dramatically across implementations, educational contexts, and student populations. Additional affective outcomes assessed by individual studies showed mixed results: contradictory findings for communication competency, promising single‐study evidence for emotional intelligence enhancement, superior stress reduction despite higher baseline stress, and reassuring findings that IVR did not significantly increase cognitive load despite the greater operational and visual complexity of the virtual environment. The limited number of studies per outcome, combined with substantial heterogeneity, constrains our ability to draw definitive conclusions about IVR’s affective impact. These findings indicate that IVR’s impact on affective outcomes is construct‐specific and implementation‐dependent, highlighting the need for standardized assessment instruments and implementation protocols to achieve consistent results in this domain.

### 4.1. Strengths and Limitations

This study contributes methodological rigor to the field by exclusively including RCTs and focusing specifically on nursing student populations, providing more robust causal inferences than previous syntheses combining diverse study designs and mixed populations. The application of Bloom’s taxonomy as a conceptual framework enabled systematic categorization of outcomes across cognitive, psychomotor, and affective domains, thereby facilitating comprehensive interpretation of IVR’s educational impact. Subgroup analyses, while limited by available data, suggested potential patterns regarding debriefing presence and assessment timing that warrant further investigation.

However, several limitations must be acknowledged. The substantial heterogeneity observed across studies reflects variations in IVR technology, intervention duration, outcome measurement, and educational contexts that may limit generalizability. Sensitivity analyses revealed instability in specific outcomes, such as knowledge acquisition and self‐efficacy, warranting cautious interpretation when applying these findings. The limited number of studies examining certain outcomes constrains effect estimate precision and may have reduced statistical power to detect meaningful differences. Moreover, most studies employed short‐term follow‐up periods, limiting understanding of long‐term educational impact. Additionally, inadequate reporting of intervention characteristics across studies restricted subgroup analyses to only debriefing presence and assessment timing, preventing comprehensive exploration of which specific intervention components drive effectiveness. Future research should prioritize standardized outcome measures, extended follow‐up assessments, detailed intervention reporting, and systematic evaluation of implementation strategies to better define IVR implementation protocols and optimize its effectiveness in nursing education.

## 5. Conclusion

This systematic review and meta‐analysis provides important evidence that IVR demonstrates significant benefits for nursing students’ knowledge acquisition and self‐efficacy, while showing limited effectiveness for skill development and mixed results for other affective outcomes. The domain‐specific effectiveness patterns challenge assumptions about universal technology benefits and indicate that IVR may be most effective for cognitive learning objectives. Our findings highlight the importance of debriefing in maximizing IVR effectiveness and reveal the transient nature of knowledge gains over time, suggesting the need for reinforcement strategies to sustain learning benefits. For educators, these results support incorporating IVR as a complementary tool for cognitive learning while maintaining traditional hands‐on approaches for psychomotor skill development. Although adverse effects were reported, they were generally mild, consisting primarily of dizziness, nausea, and visual fatigue. However, the substantial heterogeneity observed across studies reveals that the field requires standardization of technologies, protocols, and outcome measures to optimize educational impact. Future research should prioritize developing standardized implementation protocols, validated outcome measures, and longitudinal assessments to better define IVR’s role in preparing nursing students for increasingly complex healthcare environments.

## Author Contributions

Xia Li: conceptualization, methodology, investigation, data curation, validation, visualization, writing–original draft, and writing–review and editing. Yanling Hu: conceptualization, validation, supervision, and writing–review and editing. Hanmei Peng: investigation, data curation, validation, and writing–review and editing. Shiqi Luo: conceptualization, data curation, and writing–original draft. Chunsong Yang: conceptualization, data curation, and writing–review and editing. Biru Luo: supervision, project administration, and writing–review and editing. Xingli Wan: conceptualization, validation, project administration, supervision, writing–original draft, and writing–review and editing. Yuan Li: conceptualization, data curation, software, data analysis, validation, funding acquisition, supervision, writing–original draft, and writing–review and editing.

## Funding

This work was supported by the Sichuan University West China Second Hospital Teaching Reform Project (grant number: YB20250001), the Sichuan Province Nursing Association (grant number: H23020), the Sichuan Medical Association (grant number: Q2024011), the Chengdu Municipal Bureau of Science and Technology (grant number: 2024‐YF05‐00503‐SN), and the Sichuan Provincial Department of Education (grant number: JG2024‐0082).

## Disclosure

The funders had no role in study design, data collection and analysis, decision to publish, or preparation of the manuscript.

## Ethics Statement

No ethics approval was needed for this systematic review and meta‐analysis.

## Conflicts of Interest

The authors declare no conflicts of interest.

## Supporting Information

Additional supporting information can be found online in the Supporting Information section.

## Supporting information


**Supporting Information** Supporting File 1. PRISMA checklist. Supporting File 2. Search strategy for each database. Supporting Table 1. GRADE Evidence Profile for Primary Outcomes. Supporting Figure 1. Risk of bias assessment for included studies: objective outcomes. Supporting Figure 2. Risk of bias assessment for included studies: subjective outcomes. Supporting Figure 3. Sensitivity analysis for knowledge acquisition outcomes. Supporting Figure 4. Funnel plot for knowledge acquisition outcomes. Supporting Figure 5. Subgroup analysis of knowledge acquisition outcomes by debriefing presence. Supporting Figure 6. Subgroup analysis of knowledge acquisition outcomes by follow‐up timing. Supporting Figure 7. Sensitivity analysis for skill performance outcomes. Supporting Figure 8. Funnel plot for skill performance outcomes. Supporting Figure 9. Subgroup analysis of skill performance outcomes by debriefing presence. Supporting Figure 10. Subgroup analysis of skill performance outcomes by follow‐up timing. Supporting Figure 11. Forest plot for satisfaction outcomes. Supporting Figure 12. Forest plot for confidence outcomes. Supporting Figure 13. Forest plot for self‐efficacy outcomes. Supporting Figure 14. Sensitivity analysis for satisfaction outcomes. Supporting Figure 15. Sensitivity analysis for confidence outcomes. Supporting Figure 16. Sensitivity analysis for self‐efficacy outcomes.

## Data Availability

The data that support the findings of this study are available from the corresponding author upon reasonable request.

## References

[1] World Health Organization , State of the World’s Nursing 2025: Investing in Education, Jobs, Leadership and Service Delivery, 2025, World Health Organization, Geneva.

[2] Wong F. K. and Zhao Y. , Nursing Education in China: Past, Present and Future, Journal of Nursing Management. (January 2012) 20, no. 1, 38–44, 10.1111/j.1365-2834.2011.01335.x, 2-s2.0-84855552300. 22229899

[3] Jarvelainen M. , Cooper S. , and Jones J. , Nursing Students’ Educational Experience in Regional Australia: Reflections on Acute Events. A Qualitative Review of Clinical Incidents, Nurse Education in Practice. (July 2018) 31, 188–193, 10.1016/j.nepr.2018.06.007, 2-s2.0-85048999356. 29957543

[4] Horsfall J. , Cleary M. , and Hunt G. E. , Developing a Pedagogy for Nursing Teaching-Learning, Nurse Education Today. (November 2012) 32, no. 8, 930–933, 10.1016/j.nedt.2011.10.022, 2-s2.0-84868301306. 22100421

[5] Quah T. C. S. , Lau Y. , Ang W. W. , and Lau S. T. , Experiences of Immersive Virtual Reality in Healthcare Clinical Training for Nursing and Allied Health Students: A Mixed Studies Systematic Review, Nurse Education Today. (May 2025) 148, 10.1016/j.nedt.2025.106625. 39965296

[6] Ahmadi S. , Abdi A. , Nazarianpirdosti M. , Rajati F. , Rahmati M. , and Abdi A. , Challenges of Clinical Nursing Training Through Internship Approach: A Qualitative Study, Journal of Multidisciplinary Healthcare. (2020) 13, 891–900, 10.2147/jmdh.S258112. 32982265 PMC7490099

[7] Tan K. Z. Y. , Seah B. , Wong L. F. , Lee C. C. S. , Goh H. S. , and Liaw S. Y. , Simulation-Based Mastery Learning to Facilitate Transition to Nursing Practice, Nurse Educator. (November 2022) 47, no. 6, 336–341, 10.1097/nne.0000000000001224. 35667017

[8] Motola I. , Devine L. A. , Chung H. S. , Sullivan J. E. , and Issenberg S. B. , Simulation in Healthcare Education: A Best Evidence Practical Guide. Amee Guide No. 82, Medical Teacher. (October 2013) 35, no. 10, e1511–e1530, 10.3109/0142159x.2013.818632, 2-s2.0-84884516515. 23941678

[9] Shorey S. and Ng E. D. , The Use of Virtual Reality Simulation Among Nursing Students and Registered Nurses: A Systematic Review, Nurse Education Today. (2021) 98, 10.1016/j.nedt.2020.104662. 33203545

[10] Bruno R. R. , Wolff G. , Wernly B. et al., Virtual and Augmented Reality in Critical Care Medicine: The Patient’s, Clinician’s, and Researcher’s Perspective, Critical Care. (October 2022) 26, no. 1, 10.1186/s13054-022-04202-x. PMC9593998 36284350

[11] Foronda C. L. , Fernandez-Burgos M. , Nadeau C. , Kelley C. N. , and Henry M. N. , Virtual Simulation in Nursing Education: A Systematic Review Spanning 1996 to 2018, Simulation in Healthcare. (February 2020) 15, no. 1, 46–54, 10.1097/sih.0000000000000411. 32028447

[12] Pellas N. , Mystakidis S. , and Kazanidis I. , Immersive Virtual Reality in K-12 and Higher Education: A Systematic Review of the Last Decade Scientific Literature, Virtual Reality. (2021) 25, no. 3, 835–861, 10.1007/s10055-020-00489-9.

[13] Hsieh J. Y. , Lin P. C. , Sun W. N. , Lin T. R. , Kuo C. C. , and Hsu H. T. , Effectiveness of Immersive Virtual Reality in Nursing Education for Nursing Students and Nursing Staffs: A Systematic Review and Meta-Analysis, Nurse Education Today. (2025) 151, 10.1016/j.nedt.2025.106725. 40215711

[14] Ghaednia H. , Fourman M. S. , Lans A. et al., Augmented and Virtual Reality in Spine Surgery, Current Applications and Future Potentials, The Spine Journal. (October 2021) 21, no. 10, 1617–1625, 10.1016/j.spinee.2021.03.018. 33774210

[15] Salatino A. , Zavattaro C. , Gammeri R. et al., Virtual Reality Rehabilitation for Unilateral Spatial Neglect: A Systematic Review of Immersive, Semi-Immersive and Non-Immersive Techniques, Neuroscience & Biobehavioral Reviews. (September 2023) 152, 10.1016/j.neubiorev.2023.105248. 37247829

[16] Vogelsang L. , Wright S. , Risling T. et al., Exploring the Use of Immersive Virtual Reality in Nursing Education: A Scoping Review, Clinical Simulation in Nursing. (2024) 97, https://www.sciencedirect.com/science/article/pii/S1876139924001403, 10.1016/j.ecns.2024.101648.

[17] Dragnes B. , Lone A. M. S.-J. , Jensen T. H. , and Aarkrog V. , Enhancing Nursing Students’ Self-Reported Self-Efficacy and Professional Competence in Basic Life Support: The Role of Virtual Simulation Prior to High-Fidelity Training, Teaching and Learning in Nursing. (2025) 20, no. 1, e236–e243, https://www.sciencedirect.com/science/article/pii/S1557308724002269, 10.1016/j.teln.2024.10.020.

[18] Plotzky C. , Loessl B. , Kuhnert B. et al., My Hands are Running Away-Learning a Complex Nursing Skill via Virtual Reality Simulation: A Randomised Mixed Methods Study, BMC Nursing. (June 2023) 22, no. 1, 10.1186/s12912-023-01384-9. PMC10294322 37370124

[19] Liao S. , Tan M. , Chong M. , Xiong W. , Wang J. , and Luo B. , The Use of Virtual Reality to Improve Disaster Preparedness Among Nursing Students: A Randomized Study, Journal of Nursing Education. (February 2022) 61, no. 2, 93–96, 10.3928/01484834-20211213-05. 35112954

[20] Kim J. , Song J.-H. , and Ha Y.-Ok , Effects of Virtual Reality Cardiopulmonary Resuscitation Practice on the Knowledge, Skills, and Attitudes of Nursing Students: A Single-Blind Randomized Controlled Trial (Rct), Res Community Public Health Nurs. (2024) 35, no. 4, 415–423, 10.12799/rcphn.2024.00689.

[21] Liu J. Y. W. , Yin Y. H. , Kor P. P. K. et al., The Effects of Immersive Virtual Reality Applications on Enhancing the Learning Outcomes of Undergraduate Health Care Students: Systematic Review with Meta-Synthesis, Journal of Medical Internet Research. (March 2023) 25, 10.2196/39989. PMC10028520 36877550

[22] Adams N. E. , Bloom’s Taxonomy of Cognitive Learning Objectives, Journal of the Medical Library Association. (July 2015) 103, no. 3, 152–153, 10.3163/1536-5050.103.3.010, 2-s2.0-84939123648. 26213509 PMC4511057

[23] Anderson L. W. , Krathwohl D. R. , and Bloom B. S. , A Taxonomy for Learning, Teaching, and Assessing: A Revision of Bloom’s Taxonomy of Educational Objectives. (2000) european legacy.

[24] Bloom B. S. , Taxonomy of Educational Objectives: The Classification of Educational Goals, 1st Ed, Taxonomy of Educational Objectives: The Classification of Educational Goals. (1956) Longman Group, Harlow, Essex, England.

[25] Page M. J. , McKenzie J. E. , Bossuyt P. M. et al., The Prisma 2020 Statement: An Updated Guideline for Reporting Systematic Reviews, BMJ. (2021) 372, https://www.bmj.com/content/bmj/372/bmj.n71.full.pdf, 10.1136/bmj.n71. PMC8005924 33782057

[26] Sterne J. A. C. , Savović J. , Page M. J. et al., Rob 2: A Revised Tool for Assessing Risk of Bias in Randomised Trials, BMJ. (August 2019) 366, 10.1136/bmj.l4898, 2-s2.0-85071628750. 31462531

[27] Cohen J. , Statistical Power Analysis for the Behavioral Sciences, 1988, Lawrence Erlbaum Associates, Hillsdale, NJ.

[28] Fu R. , Gartlehner G. , Grant M. et al., Conducting Quantitative Synthesis when Comparing Medical Interventions: Ahrq and the Effective Health Care Program, Journal of Clinical Epidemiology. (November 2011) 64, no. 11, 1187–1197, 10.1016/j.jclinepi.2010.08.010, 2-s2.0-80053351531. 21477993

[29] Aydın Doğan R. and Yazıcı S. , Use and Effectiveness of Innovative Virtual Reality Application in Teaching Fetal Development: A Randomized Controlled Trial, Comput Inform Nurs. (July 2024) 42, no. 7, 515–521, 10.1097/cin.0000000000001036. 38453431

[30] Babaita A. O. , Kako M. , Teramoto C. et al., Face-To-Face Versus 360° Vr Video: A Comparative Study of Two Teaching Methods in Nursing Education, BMC Nursing. (March 2024) 23, no. 1, 10.1186/s12912-024-01866-4. PMC10962166 38523319

[31] Bani S. , Ayman K. , Malak M. Z. , El-Qirem F. A. , Alhussami M. , and El-hneiti M. , Effect of Virtual Reality Simulation as a Teaching Strategy on Nursing Students’ Satisfaction, Self-Confidence, Performance, and Physiological Measures in Jordan, Teaching and Learning in Nursing. (2024) 19, no. 1, e235–e241, https://www.sciencedirect.com/science/article/pii/S1557308723002299, 10.1016/j.teln.2023.11.005.

[32] Berg H. and Steinsbekk A. , Is Individual Practice in an Immersive and Interactive Virtual Reality Application Non-Inferior to Practicing with Traditional Equipment in Learning Systematic Clinical Observation? A Randomized Controlled Trial, BMC Medical Education. (April 2020) 20, no. 1, 10.1186/s12909-020-02030-7. PMC7181571 32326948

[33] Berg H. and Steinsbekk A. , The Effect of Self-Practicing Systematic Clinical Observations in a Multiplayer, Immersive, Interactive Virtual Reality Application Versus Physical Equipment: A Randomized Controlled Trial, Advances in Health Sciences Education: Theory and Practice. (May 2021) 26, no. 2, 667–682, 10.1007/s10459-020-10019-6. 33511505 PMC8041677

[34] Chao Y. C. , Hu S. H. , Chiu H. Y. , Huang P. H. , Tsai H. T. , and Chuang Y. H. , The Effects of an Immersive 3d Interactive Video Program on Improving Student Nurses’ Nursing Skill Competence: A Randomized Controlled Trial Study, Nurse Education Today. (August 2021) 103, 10.1016/j.nedt.2021.104979. 34049120

[35] Chen C. J. , Chen Y. C. , Lee M. Y. , Wang C. H. , and Sung H. C. , Effects of Three-Dimensional Holograms on the Academic Performance of Nursing Students in a Health Assessment and Practice Course: A pretest-intervention-posttest Study, Nurse Education Today. (November 2021) 106, 10.1016/j.nedt.2021.105081. 34418588

[36] Chou C. H. , Tai H. C. , and Chen S. L. , The Effects of Introducing Virtual Reality Communication Simulation in Students’ Learning in a Fundamentals of Nursing Practicum: A Pragmatic Randomized Control Trials, Nurse Education in Practice. (January 2024) 74, 10.1016/j.nepr.2023.103837. 38006647

[37] Gray M. , Downer T. , Hartz D. , Andersen P. , Hanson J. , and Gao Y. , The Impact of Three-Dimensional Visualisation on Midwifery Student Learning, Compared with Traditional Education for Teaching the Third Stage of Labour: A Pilot Randomised Controlled Trial, Nurse Education Today. (January 2022) 108, 10.1016/j.nedt.2021.105184. 34717099

[38] Jeong S. and Cha C. , Effects of Immersive Virtual Reality Simulation–Based Maternity Nursing Education: A Randomized Controlled Trial, Clinical Simulation in Nursing. (2024) 97, https://www.sciencedirect.com/science/article/pii/S1876139924001233, 10.1016/j.ecns.2024.101631.

[39] Lee J. J. , Tsang V. W. Y. , Chan M. M. K. et al., Virtual Reality Simulation-Enhanced Blood Transfusion Education for Undergraduate Nursing Students: A Randomised Controlled Trial, Nurse Education Today. (October 2023) 129, 10.1016/j.nedt.2023.105903. 37467707

[40] Lee M. , Kim S. K. , Go Y. , Jeong H. , and Lee Y. , Positioning Virtual Reality as Means of Clinical Experience in Mental Health Nursing Education: A Quasi-Experimental Study, Applied Nursing Research. (June 2024) 77, 10.1016/j.apnr.2024.151800. 38796255

[41] Lo Y. T. , Yang C. C. , Yeh T. F. , Tu H. Y. , and Chang Y. C. , Effectiveness of Immersive Virtual Reality Training in Nasogastric Tube Feeding Education: A Randomized Controlled Trial, Nurse Education Today. (December 2022) 119, 10.1016/j.nedt.2022.105601. 36244254

[42] Mayor Silva L. I. , Caballero de la Calle R. , Cuevas-Budhart M. A. , Martin Martin J. O. , Blanco Rodriguez J. M. , and Gómez Del Pulgar García Madrid M. , Development of Communication Skills Through Virtual Reality on Nursing School Students: Clinical Trial, Comput Inform Nurs. (January 2023) 41, no. 1, 24–30, 10.1097/cin.0000000000000866. 35363632

[43] Rasouli B. , The Effect of Virtual Reality-Based Teaching (Vrbt) on Nursing Students’ Learning Performance and Engagement in Anatomy, Journal of Medical Education. (2025) 23, no. 1, 10.5812/jme-151030.

[44] Zahran H. , Malak M. Z. , El-Qirem F. , and Asfour B. , The Effect of Virtual Reality Airway Management as a Learning Strategy on Performance, Self-Efficacy, and Emotional Intelligence Among Nursing Students in the West Bank/Palestine, Teaching and Learning in Nursing. (2025) 20, no. 1, e35–e42, https://www.sciencedirect.com/science/article/pii/S1557308724001616, 10.1016/j.teln.2024.07.023.

[45] World Bank Country Classifications by Income Level for 2024-2025, 2024, https://blogs.worldbank.org/en/opendata/world-bank-country-classifications-by-income-level-for-2024-2025.

[46] O′Brien B. C. and Battista A. , Situated Learning Theory in Health Professions Education Research: A Scoping Review, Advances in Health Sciences Education: Theory and Practice. (May 2020) 25, no. 2, 483–509, 10.1007/s10459-019-09900-w, 2-s2.0-85068157790. 31230163

[47] Brandon A. F. and All A. C. , Constructivism Theory Analysis and Application to Curricula, Nursing Education Perspectives. (April 2010) 31, no. 2, 89–92. 20455364

[48] Watts P. I. , Kelly R. , Bowler F. et al., Onward and Upward: Introducing the Healthcare Simulation Standards of Best Practicetm, Clinical Simulation in Nursing. (2021) 58, 1–4, https://www.sciencedirect.com/science/article/pii/S1876139921000931, 10.1016/j.ecns.2021.08.006.

[49] Zhang H. , Mörelius E. , Goh S. H. L. , and Wang W. , Effectiveness of Video-Assisted Debriefing in Simulation-Based Health Professions Education: A Systematic Review of Quantitative Evidence, Nurse Educator. (June 2019) 44, no. 3, E1–e6, 10.1097/nne.0000000000000562, 2-s2.0-85060517793. 30015683

[50] Sahin Karaduman G. , Basak T. , and Duman S. , Three Debriefing Methods in Virtual Patient Simulation: A Randomized Controlled Trial, Nurse Education in Practice. (August 2025) 87, 10.1016/j.nepr.2025.104447. 40617131

[51] Secheresse T. , Lima L. , and Pansu P. , Focusing on Explicit Debriefing for Novice Learners in Healthcare Simulations: A Randomized Prospective Study, Nurse Education in Practice. (February 2021) 51, 10.1016/j.nepr.2020.102914. 33323287

[52] Ruchkin D. S. , Grafman J. , Cameron K. , and Berndt R. S. , Working Memory Retention Systems: A State of Activated Long-Term Memory, Behavioral and Brain Sciences. (December 2003) 26, no. 6, 709–728, 10.1017/s0140525x03000165. 15377128

[53] Custers E. J. and Ten Cate O. T. , Very Long-Term Retention of Basic Science Knowledge in Doctors After Graduation, Medical Education. (April 2011) 45, no. 4, 422–430, 10.1111/j.1365-2923.2010.03889.x, 2-s2.0-79952637491. 21401691

