# Supplementary material for: Effects of Immersive Virtual Reality Simulation on Nursing Students’ Learning Outcomes: A Systematic Review and Meta‐Analysis
Source: J Nurs Manag. 2026 May 15;2026:4686851. doi: 10.1155/jonm/4686851 (PMC13179422; doi:10.1155/jonm/4686851)
Supplement: Supplementary file 1 — Supporting Information Supporting File 1. PRISMA checklist. Supporting File 2. Search strategy for each database. Supporting Table 1. GRADE Evidence Profile for Primary Outcomes. Supporting Figure 1. Risk of bias assessment for included studies: objective outcomes. Supporting Figure 2. Risk of bias assessment for included studies: subjective outcomes. Supporting Figure 3. Sensitivity analysis for knowledge acquisition outcomes. Supporting Figure 4. Funnel plot for knowledge acquisition outcomes. Supporting Figure 5. Subgroup analysis of knowledge acquisition outcomes by debriefing presence. Supporting Figure 6. Subgroup analysis of knowledge acquisition outcomes by follow‐up timing. Supporting Figure 7. Sensitivity analysis for skill performance outcomes. Supporting Figure 8. Funnel plot for skill performance outcomes. Supporting Figure 9. Subgroup analysis of skill performance outcomes by debriefing presence. Supporting Figure 10. Subgroup analysis of skill performance outcomes by follow‐up timing. Supporting Figure 11. Forest plot for satisfaction outcomes. Supporting Figure 12. Forest plot for confidence outcomes. Supporting Figure 13. Forest plot for self‐efficacy outcomes. Supporting Figure 14. Sensitivity analysis for satisfaction outcomes. Supporting Figure 15. Sensitivity analysis for confidence outcomes. Supporting Figure 16. Sensitivity analysis for self‐efficacy outcomes. [file JONM-2026-4686851-s001.docx]

**Supplementary Materials**

[Supporting File 1. PRISMA checklist 1](#_Toc216012842)

[Supporting File 2. Search strategy for each database (Updated on 25 March, 2025) 7](#_Toc216012843)

[MEDLINE (Ovid) 7](#_Toc216012844)

[Embase (Ovid) 8](#_Toc216012845)

[CINAHL (EBSCOhost) 10](#_Toc216012846)

[Cochrane Central Register of Controlled Trials (Cochrane Library) 12](#_Toc216012847)

[Supporting Table 1. GRADE Evidence Profile for Primary Outcomes. 13](#_Toc216012848)

[Supporting Figure 1. Risk of bias assessment for included studies: objective outcomes. 14](#_Toc216012849)

[Supporting Figure 2. Risk of bias assessment for included studies: subjective outcomes. 14](#_Toc216012850)

[Supporting Figure 3. Sensitivity analysis for knowledge acquisition outcomes 15](#_Toc216012851)

[Supporting Figure 4. Funnel plot for knowledge acquisition outcomes 16](#_Toc216012852)

[Supporting Figure 5. Subgroup analysis of knowledge acquisition outcomes by debriefing presence 17](#_Toc216012853)

[Supporting Figure 6. Subgroup analysis of knowledge acquisition outcomes by follow-up timing 18](#_Toc216012854)

[Supporting Figure 7. Sensitivity analysis for skill performance outcomes 19](#_Toc216012855)

[Supporting Figure 8. Funnel plot for skill performance outcomes 20](#_Toc216012856)

[Supporting Figure 9. Subgroup analysis of skill performance outcomes by debriefing presence 21](#_Toc216012857)

[Supporting Figure 10. Subgroup analysis of skill performance outcomes by follow-up timing 22](#_Toc216012858)

[Supporting Figure 11. Forest plot for satisfaction outcomes 22](#_Toc216012859)

[Supporting Figure 12. Forest plot for confidence outcomes 23](#_Toc216012860)

[Supporting Figure 13. Forest plot for self-efficacy outcomes 23](#_Toc216012861)

[Supporting Figure 14. Sensitivity analysis for satisfaction outcomes 24](#_Toc216012862)

[Supporting Figure 15. Sensitivity analysis for confidence outcomes 25](#_Toc216012863)

[Supporting Figure 16. Sensitivity analysis for self-efficacy outcomes 26](#_Toc216012864)

**Supporting File 1.** PRISMA checklist

|  | Item Description | Location (or reason for not reporting) |
| --- | --- | --- |
| **Title and Abstract** |  |  |
| [1. Title](https:/resources.equator-network.org/reporting-guidelines/prisma/items/title.html?utm_source=prisma&utm_medium=checklist&utm_campaign=1_1) | Identify the report as a systematic review. | Title |
| [2. Abstract](https:/resources.equator-network.org/reporting-guidelines/prisma/items/abstract.html?utm_source=prisma&utm_medium=checklist&utm_campaign=1_1) | Include all items from the *PRISMA 2020 for Abstracts* checklist. | Abstract |
| **Introduction** |  |  |
| [3. Rationale](https:/resources.equator-network.org/reporting-guidelines/prisma/items/rationale.html?utm_source=prisma&utm_medium=checklist&utm_campaign=1_1) | Describe the rationale for the review in the context of existing knowledge. | 1. Introduction  paragraph 1-5 |
| [4. Objectives](https:/resources.equator-network.org/reporting-guidelines/prisma/items/objectives.html?utm_source=prisma&utm_medium=checklist&utm_campaign=1_1) | Provide an explicit statement of the objective(s) or question(s) the review addresses. | 1. Introduction  paragraph 6 |
| **Methods** |  |  |
| [5. Eligibility criteria](https:/resources.equator-network.org/reporting-guidelines/prisma/items/eligibility-criteria.html?utm_source=prisma&utm_medium=checklist&utm_campaign=1_1) | Specify the inclusion and exclusion criteria for the review and how studies were grouped for the syntheses. | 2.3 Eligibility criteria  Paragraph 1-2 |
| [6. Information sources](https:/resources.equator-network.org/reporting-guidelines/prisma/items/information-sources.html?utm_source=prisma&utm_medium=checklist&utm_campaign=1_1) | Specify all databases, registers, websites, organisations, reference lists and other sources searched or consulted to identify studies. Specify the date when each source was last searched or consulted. | 2.2 Search strategy  Paragraph 1 |
| [7. Search](https:/resources.equator-network.org/reporting-guidelines/prisma/items/search.html?utm_source=prisma&utm_medium=checklist&utm_campaign=1_1) | Present the full search strategies for all databases, registers and websites, including any filters and limits used. | Supporting File 1 |
| [8. Selection Process](https:/resources.equator-network.org/reporting-guidelines/prisma/items/selection-process.html?utm_source=prisma&utm_medium=checklist&utm_campaign=1_1) | Specify the methods used to decide whether a study met the inclusion criteria of the review, including how many reviewers screened each record and each report retrieved, whether they worked independently, and, if applicable, details of automation tools used in the process. | 2.4 Study selection and data extraction  Paragraph 1 |
| [9. Data collection process](https:/resources.equator-network.org/reporting-guidelines/prisma/items/data-collection-process.html?utm_source=prisma&utm_medium=checklist&utm_campaign=1_1) | Specify the methods used to collect data from reports, including how many reviewers collected data from each report, whether they worked independently, any processes for obtaining or confirming data from study investigators, and if applicable, details of automation tools used in the process. | 2.4 Study selection and data extraction  Paragraph 1 |
| 10. Data Items |  |  |
| [10a. Outcomes](https:/resources.equator-network.org/reporting-guidelines/prisma/items/data-items-outcomes.html?utm_source=prisma&utm_medium=checklist&utm_campaign=1_1) | List and define all outcomes for which data were sought. Specify whether all results that were compatible with each outcome domain in each study were sought (e.g. for all measures, time points, analyses), and if not, the methods used to decide which results to collect. | 2.3 Eligibility criteria  Paragraph 1 |
| [10b. Other Variables](https:/resources.equator-network.org/reporting-guidelines/prisma/items/data-items-other-variables.html?utm_source=prisma&utm_medium=checklist&utm_campaign=1_1) | List and define all other variables for which data were sought (e.g. participant and intervention characteristics, funding sources). Describe any assumptions made about any missing or unclear information. | 2.4 Study selection and data extraction  Paragraph 1 |
| [11. Risk of bias in individual studies](https:/resources.equator-network.org/reporting-guidelines/prisma/items/risk-of-bias-in-individual-studies.html?utm_source=prisma&utm_medium=checklist&utm_campaign=1_1) | Specify the methods used to assess risk of bias in the included studies, including details of the tool(s) used, how many reviewers assessed each study and whether they worked independently, and if applicable, details of automation tools used in the process. | 2.5 Risk of bias assessment  Paragraph 1 |
| [12. Effect measures](https:/resources.equator-network.org/reporting-guidelines/prisma/items/effect-measures.html?utm_source=prisma&utm_medium=checklist&utm_campaign=1_1) | Specify for each outcome the effect measure(s) (e.g. risk ratio, mean difference) used in the synthesis or presentation of results. | 2.6 Statistical analysis  Paragraph 1 |
| 13. Synthesis Methods |  |  |
| [13a. Deciding which studies were eligible for each synthesis](https:/resources.equator-network.org/reporting-guidelines/prisma/items/synthesis-methods-eligibility.html?utm_source=prisma&utm_medium=checklist&utm_campaign=1_1) | Describe the processes used to decide which studies were eligible for each synthesis (such as tabulating the study intervention characteristics and comparing against the planned groups for each synthesis described in item 5. | 2.3 Eligibility criteria  2.4 Study selection and data extraction |
| [13b. Data preparation methods](https:/resources.equator-network.org/reporting-guidelines/prisma/items/synthesis-methods-data-preparation.html?utm_source=prisma&utm_medium=checklist&utm_campaign=1_1) | Describe any methods required to prepare the data for presentation or synthesis, such as handling of missing summary statistics, or data conversions. | 2.6 Statistical analysis |
| [13c. Methods for tabulating or displaying results](https:/resources.equator-network.org/reporting-guidelines/prisma/items/synthesis-methods-tabulating-or-displaying-results.html?utm_source=prisma&utm_medium=checklist&utm_campaign=1_1) | Describe any methods used to tabulate or visually display results of individual studies and syntheses. | 2.6 Statistical analysis |
| [13d. Synthesis methods](https:/resources.equator-network.org/reporting-guidelines/prisma/items/synthesis-methods-synthesis-methods.html?utm_source=prisma&utm_medium=checklist&utm_campaign=1_1) | Describe any methods used to synthesize results and provide a rationale for the choice(s). If meta-analysis was performed, describe the model(s), method(s) to identify the presence and extent of statistical heterogeneity, and software package(s) used. | 2.6 Statistical analysis |
| [13e. Methods for exploring heterogeneity](https:/resources.equator-network.org/reporting-guidelines/prisma/items/synthesis-methods-exploring-heterogeneity.html?utm_source=prisma&utm_medium=checklist&utm_campaign=1_1) | Describe any methods used to explore possible causes of heterogeneity among study results (e.g. subgroup analysis, meta-regression). | 2.6 Statistical analysis |
| [13f. Sensitivity analyses](https:/resources.equator-network.org/reporting-guidelines/prisma/items/synthesis-methods-sensitivity-analyses.html?utm_source=prisma&utm_medium=checklist&utm_campaign=1_1) | Describe any sensitivity analyses conducted to assess robustness of the synthesized results. | 2.6 Statistical analysis |
| [14. Reporting bias assessment](https:/resources.equator-network.org/reporting-guidelines/prisma/items/reporting-bias-assessment.html?utm_source=prisma&utm_medium=checklist&utm_campaign=1_1) | Describe any methods used to assess risk of bias due to missing results in a synthesis (arising from reporting biases). | 2.5 Risk of bias assessment and certainty of evidence evaluation |
| [15. Certainty assessment](https:/resources.equator-network.org/reporting-guidelines/prisma/items/certainty-assessment.html?utm_source=prisma&utm_medium=checklist&utm_campaign=1_1) | Describe any methods used to assess certainty (or confidence) in the body of evidence for an outcome. | 2.5 Risk of bias assessment and certainty of evidence evaluation |
| **Results** |  |  |
| 16. Study Selection |  |  |
| [16a. Results of the search and selection process](https:/resources.equator-network.org/reporting-guidelines/prisma/items/study-selection-search-results.html?utm_source=prisma&utm_medium=checklist&utm_campaign=1_1) | Describe the results of the search and selection process, from the number of records identified in the search to the number of studies included in the review, ideally using a flow diagram. | Figure 1. |
| [16b. Excluded studies](https:/resources.equator-network.org/reporting-guidelines/prisma/items/study-selection-excluded-studies.html?utm_source=prisma&utm_medium=checklist&utm_campaign=1_1) | Cite studies that might appear to meet the inclusion criteria, but which were excluded, and explain why they were excluded. | Figure 1. |
| [17. Study characteristics](https:/resources.equator-network.org/reporting-guidelines/prisma/items/study-characteristics.html?utm_source=prisma&utm_medium=checklist&utm_campaign=1_1) | Cite each included study and present its characteristics. | Table 1. |
| [18. Risk of bias in studies](https:/resources.equator-network.org/reporting-guidelines/prisma/items/risk-of-bias-in-studies.html?utm_source=prisma&utm_medium=checklist&utm_campaign=1_1) | Present assessments of risk of bias for each included study. | Supporting Figure 1 & 2 |
| [19. Results of individual studies](https:/resources.equator-network.org/reporting-guidelines/prisma/items/results-of-individual-studies.html?utm_source=prisma&utm_medium=checklist&utm_campaign=1_1) | For all outcomes, present, for each study: (a) summary statistics for each group (where appropriate) and (b) an effect estimate and its precision (e.g. confidence/credible interval), ideally using structured tables or plots. | Table 1 |
| 20. Results of Synthesis |  |  |
| [20a. Summary of studies](https:/resources.equator-network.org/reporting-guidelines/prisma/items/results-of-syntheses-summary-of-studies.html?utm_source=prisma&utm_medium=checklist&utm_campaign=1_1) | For each synthesis, briefly summarise the characteristics and risk of bias among contributing studies. | 3.1 Study characteristics  Paragraph 1-2  3.2 Risk of bias of included studies  Paragraph 1 |
| [20b. Statistical results](https:/resources.equator-network.org/reporting-guidelines/prisma/items/results-of-syntheses-statistical-results.html?utm_source=prisma&utm_medium=checklist&utm_campaign=1_1) | Present results of all statistical syntheses conducted. If meta-analysis was done, present for each the summary estimate and its precision (e.g. confidence/credible interval) and measures of statistical heterogeneity. If comparing groups, describe the direction of the effect. | 3.3.1 Primary outcomes: knowledge  Paragraph 1-2  3.3.2 Primary outcomes: skill performance  Paragraph 1-2  3.3.3 Secondary outcomes: Affective domain  Paragraph 2  Figure 2 & 3  Supporting Figure 5-6, 9-13 |
| [20c. Heterogeneity](https:/resources.equator-network.org/reporting-guidelines/prisma/items/results-of-syntheses-heterogeneity.html?utm_source=prisma&utm_medium=checklist&utm_campaign=1_1) | Present results of all investigations of possible causes of heterogeneity among study results. | 3.3.1 Primary outcomes: knowledge  Paragraph 1-2  3.3.2 Primary outcomes: skill performance  Paragraph 1-2  3.3.3 Secondary outcomes: Affective domain  Paragraph 2  Figure 2 & 3  Supporting Figure 5-6, 9-13 |
| [20d. Sensitivity analyses](https:/resources.equator-network.org/reporting-guidelines/prisma/items/results-of-syntheses-sensitivity-analyses.html?utm_source=prisma&utm_medium=checklist&utm_campaign=1_1) | Present results of all sensitivity analyses conducted to assess the robustness of the synthesized results. | 3.3.1 Primary outcomes: knowledge  Paragraph 1  3.3.2 Primary outcomes: skill performance  Paragraph 1  3.3.3 Secondary outcomes: Affective domain  Paragraph 2  Supporting Figure 3, 5, 14-16 |
| [21. Risk of reporting biases in syntheses](https:/resources.equator-network.org/reporting-guidelines/prisma/items/risk-of-reporting-biases-in-syntheses.html?utm_source=prisma&utm_medium=checklist&utm_campaign=1_1) | Present assessments of risk of bias due to missing results (arising from reporting biases) for each synthesis assessed. | Supporting Figure 1&2 |
| [22. Certainty of evidence](https:/resources.equator-network.org/reporting-guidelines/prisma/items/certainty-of-evidence.html?utm_source=prisma&utm_medium=checklist&utm_campaign=1_1) | Present assessments of certainty (or confidence) in the body of evidence for each outcome assessed. | 3.2 Risk of bias of included studies and certainty of evidence |
| **Discussion** |  |  |
| 23. Discussion |  |  |
| [23a. General interpretation of the results](https:/resources.equator-network.org/reporting-guidelines/prisma/items/discussion-general-interpretation.html?utm_source=prisma&utm_medium=checklist&utm_campaign=1_1) | Provide a general interpretation of the results in the context of other evidence. | 4. Discussion  Paragraph 1 |
| [23b. Limitations of included evidence](https:/resources.equator-network.org/reporting-guidelines/prisma/items/discussion-limitations-of-included-evidence.html?utm_source=prisma&utm_medium=checklist&utm_campaign=1_1) | Discuss any limitations of the evidence included in the review. | 4.1 Strengths and limitations |
| [23c. Limitations of the review processes](https:/resources.equator-network.org/reporting-guidelines/prisma/items/discussion-limitations-of-review-process.html?utm_source=prisma&utm_medium=checklist&utm_campaign=1_1) | Discuss any limitations of the review processes used. | 4.1 Strengths and limitations |
| [23d. Implications](https:/resources.equator-network.org/reporting-guidelines/prisma/items/discussion-implications.html?utm_source=prisma&utm_medium=checklist&utm_campaign=1_1) | Discuss implications of the results for practice, policy, and future research. | 4. Discussion  Paragraph 2-5 |
| **Other Information** |  |  |
| 24. Registration and Protocol |  |  |
| [24a. Registration](https:/resources.equator-network.org/reporting-guidelines/prisma/items/registration-and-protocol-registration.html?utm_source=prisma&utm_medium=checklist&utm_campaign=1_1) | Provide registration information for the review, including register name and registration number, or state that the review was not registered. | 2.1 Study design  Paragraph 1 |
| [24b. Protocol](https:/resources.equator-network.org/reporting-guidelines/prisma/items/registration-and-protocol-protocol.html?utm_source=prisma&utm_medium=checklist&utm_campaign=1_1) | Indicate where the review protocol can be accessed, or state that a protocol was not prepared. | 2.1 Study design  Paragraph 1 |
| [24c. Amendments](https:/resources.equator-network.org/reporting-guidelines/prisma/items/registration-and-protocol-amendments.html?utm_source=prisma&utm_medium=checklist&utm_campaign=1_1) | Describe and explain any amendments to information provided at registration or in the protocol. | 2.1 Study design  Paragraph 1 |
| [25. Support](https:/resources.equator-network.org/reporting-guidelines/prisma/items/support.html?utm_source=prisma&utm_medium=checklist&utm_campaign=1_1) | Describe sources of financial or non-financial support for the review, and the role of the funders or sponsors in the review. | Funding sources |
| [26. Competing Interests](https:/resources.equator-network.org/reporting-guidelines/prisma/items/competing-interests.html?utm_source=prisma&utm_medium=checklist&utm_campaign=1_1) | Declare any competing interests of review authors. | Declaration of competing interest |
| [27. Availability of data, code, and other materials](https:/resources.equator-network.org/reporting-guidelines/prisma/items/availability-of-materials.html?utm_source=prisma&utm_medium=checklist&utm_campaign=1_1) | Report which of the following are publicly available and where they can be found: template data collection forms; data extracted from included studies; data used for all analyses; analytic code; any other materials used in the review. | Data availability |

**Supporting File 2**. Search strategy for each database (Updated on 25 March, 2025)

[MEDLINE (Ovid)](http://182.150.59.104:8888/https/77726476706e69737468656265737421ffe14898342026547d5ac7a38e5c277b58a955/ovid-new-b/ovidweb.cgi?&S=CPPCFPAOODEBCOIDIPJJNGPFPKGEAA00&Database+Field+Guide=17)

#1 Students, Nursing/ or (nurs* adj2 (pupil* or student* or undergraduate* or baccalaureate* or graduate* or trainee* or educator*)).ti,ab,kw.

#2 exp Computer Simulation/ or exp Computer-Assisted Instruction/ or exp Simulation Training/

#3 (virtual* adj2 (realit* or realis* or train* or simulat* or educat* or instruct* or teach* or mentor* or learn* or patient* or environment* or hospital or ward or system* or setting* or world)).ti,ab,kw.

#4 ((computer* or video* or patient*) adj2 simulat*).ti,ab,kw.

#5 (("computer assist*" or "computer aid*" or "computer based" or computerized or "video assist*" or "video aid*") adj2 (realit* or realis* or train* or simulat* or educat* or instruct* or self-instruct* or teach* or mentor* or learn*)).ti,ab,kw.

#6 (simulat* adj2 (realit* or realis* or train* or educat* or instruct* or teach* or mentor* or learn*)).ti,ab,kw.

#7 ((augmented or artificial) adj2 realit*).ti,ab,kw.

#8 (VR or CAI or vSim or VRMagic or avatar* or Head-mounted Display* or HMD* or Cave Automatic Virtual Environment or CAVE or Oculus Rift or HTC Vive).ti,ab,kw.

#9 or/2-8

#10 ((Randomized Controlled Trial or Controlled Clinical Trial).pt. or (randomized or randomised).ti,ab. or placebo.ti,ab. or drug therapy.fs. or randomly.ti,ab. or trial.ti,ab. or groups.ti,ab.) not ((exp animals/ or exp plants/) not humans.sh) ^[[1]](#footnote-1)^

#11 (exp Clinical Studies as Topic/ or exp Clinical Trials as Topic/ or Controlled Before-After Studies/ or Interrupted Time Series Analysis/ or ((clinical or controlled or (cross adj over) or crossover or equivalent or pragmatic or randomisation or randomised or randomization or randomized) adj3 (studies or study or trial or trials)).ti,ab,kf. or ((interrupted adj time) or (non adj (randomised or randomized)) or nonequivalent or nonrandomised or nonrandomized).ti,ab,kf. or ((one adj group) and (((post or pre) adj test) or pretest or posttest)).ti,ab,kf. or (((pretest or (pre adj5 (intervention or posttest or test))) and (posttest or (post adj5 (intervention or test)))) or (pretest adj5 posttest) or quasiexperiment* or (quasi adj experiment*) or ((single adj group) and (group adj study)) or (uncontrolled adj (studies or study))).ti,ab,kf. or intervention.ti.) not ((exp animals/ or exp plants/) not humans.sh) ^[[2]](#footnote-2)^

#12 10 or 11

#13 1 and 9 and 12

**Total: 1483**

Embase (Ovid)

#1 nursing student/ or (nurs* adj2 (pupil* or student* or undergraduate* or baccalaureate* or graduate* or trainee* or educator*)).ti,ab,kw.

#2 exp computer simulation/ or exp simulation training/

#3 (virtual* adj2 (realit* or realis* or train* or simulat* or educat* or instruct* or teach* or mentor* or learn* or patient* or environment* or hospital or ward or system* or setting* or world)).ti,ab,kw.

#4 ((computer* or video* or patient*) adj2 simulat*).ti,ab,kw.

#5 (("computer assist*" or "computer aid*" or "computer based" or computerized or "video assist*" or "video aid*") adj2 (realit* or realis* or train* or simulat* or educat* or instruct* or self-instruct* or teach* or mentor* or learn*)).ti,ab,kw.

#6 (simulat* adj2 (realit* or realis* or train* or educat* or instruct* or teach* or mentor* or learn*)).ti,ab,kw.

#7 ((augmented or artificial) adj2 realit*).ti,ab,kw.

#8 (VR or CAI or vSim or VRMagic or avatar* or Head-mounted Display* or HMD* or Cave Automatic Virtual Environment or CAVE or Oculus Rift or HTC Vive).ti,ab,kw.

#9 or/2-8

#10 exp randomized controlled trial/ or controlled clinical trial/ or randomization/ or intermethod comparison/ or double blind procedure/ or human experiment/

#11 (random$ or placebo or compare or compared or comparison).ti,ab.

#12 ((evaluated or evaluate or evaluating or assessed or assess) and (compare or compared or comparing or comparison)).ab.

#13 (open adj label).ti,ab.

#14 ((double or single or doubly or singly) adj (blind or blinded or blindly)).ti,ab.

#15 parallel group$1.ti,ab.

#16 (crossover or cross over).ti,ab.

#17 ((assign$ or match or matched or allocation) adj5 (alternate or group$1 or intervention$1 or patient$1 or subject$1 or participant$1)).ti,ab.

#18 (assigned or allocated).ti,ab.

#19 (controlled adj7 (study or design or trial)).ti,ab.

#20 (volunteer or volunteers).ti,ab.

#21 trial.ti.

#22 or/10-21

#23 (random$ adj sampl$ adj7 ("cross section$" or questionnaire$1 or survey$ or database$1)).ti,ab. not (comparative study/ or controlled study/ or randomi?ed controlled.ti,ab. or randomly assigned.ti,ab.)

#24 cross-sectional study/ not (exp randomized controlled trial/ or controlled clinical trial/ or controlled study/ or randomi?ed controlled.ti,ab. or control group$1.ti,ab.)

#25 (((case adj control$) and random$) not randomi?ed controlled).ti,ab.

#26 systematic review.ti,ab. not (trial or study).ti.

#27 (nonrandom$ not random$).ti,ab.

#28 "random field$".ti,ab.

#29 (random cluster adj3 sampl$).ti,ab.

#30 (review.ab. and review.pt.) not trial.ti.

#31 "we searched".ab. and (review.ti. or review.pt.)

#32 "update review".ab.

#33 (databases adj4 searched).ab.

#34 (rat or rats or mouse or mice or swine or porcine or murine or sheep or lambs or pigs or piglets or rabbit or rabbits or cat or cats or dog or dogs or cattle or bovine or monkey or monkeys or trout or marmoset$1).ti. and animal experiment/

#35 animal experiment/ not (human experiment/ or human/)

#36 or/23-35

#37 22 not 36 ^[[3]](#footnote-3)^

#38 (exp clinical study/ or exp clinical trial/ or ((clinical or controlled or equivalent or pragmatic) adj3 (studies or study or trial or trials)).ti,ab,kf. or ((interrupted adj time) or nonequivalent or nonrandomised or nonrandomized).ti,ab,kf. or ((one adj group) and (((post or pre) adj test) or pretest or posttest)).ti,ab,kf. or (((pretest or (pre adj5 (intervention or posttest or test))) and (posttest or (post adj5 (intervention or test)))) or (pretest adj5 posttest) or quasiexperiment* or (quasi adj experiment*) or ((single adj group) and (group adj study)) or (uncontrolled adj (studies or study))).ti,ab,kf. or intervention.ti.) not ((exp animals/ or exp plants/) not humans.sh)

#39 37 or 38

#40 1 and 9 and 39

**Total: 952**

CINAHL (EBSCOhost)

S1 MH "Students, Nursing+"

S2 TI (nurs* N2 (pupil* or student* or undergraduate* or baccalaureate* or graduate* or trainee* or educator*)) or AB (nurs* N2 (pupil* or student* or undergraduate* or baccalaureate* or graduate* or trainee* or educator*))

S3 S1 or S2

S4 MH "Computer Simulation+" or MH "Computer-Assisted Instruction+"

S5 TI (virtual* N2 (realit* or realis* or train* or simulat* or educat* or instruct* or teach* or mentor* or learn* or patient* or environment* or hospital or ward or system* or setting* or world)) or AB (virtual* N2 (realit* or realis* or train* or simulat* or educat* or instruct* or teach* or mentor* or learn* or patient* or environment* or hospital or ward or system* or setting* or world))

S6 TI ((computer* or video* or patient*) N2 simulat*) or AB ((computer* or video* or patient*) N2 simulat*)

S7 TI (("computer assist*" or "computer aid*" or "computer based" or computerized or "video assist*" or "video aid*") N2 (realit* or realis* or train* or simulat* or educat* or instruct* or self-instruct* or teach* or mentor* or learn*)) or AB (("computer assist*" or "computer aid*" or "computer based" or computerized or "video assist*" or "video aid*") N2 (realit* or realis* or train* or simulat* or educat* or instruct* or self-instruct* or teach* or mentor* or learn*))

S8 TI (simulat* N2 (realit* or realis* or train* or educat* or instruct* or teach* or mentor* or learn*)) or AB (simulat* N2 (realit* or realis* or train* or educat* or instruct* or teach* or mentor* or learn*))

S9 TI ((augmented or artificial) N2 realit*) or AB ((augmented or artificial) N2 realit*)

S10 TI (VR or CAI or vSim or VRMagic or avatar* or "Head-mounted Display*" or HMD* or "Cave Automatic Virtual Environment" or CAVE or "Oculus Rift" or HTC Vive) or AB (VR or CAI or vSim or VRMagic or avatar* or "Head-mounted Display*" or HMD* or "Cave Automatic Virtual Environment" or CAVE or "Oculus Rift" or HTC Vive)

S11 S4 or S5 or S6 or S7 or S8 or S9 or S10

S12 (MH "Clinical Trials+") or TI (RCT OR RCTs OR random* OR placebo* OR CCT OR CCTs OR "controlled trial*") OR AB (RCT OR RCTs OR random* OR placebo* OR CCT OR CCTs OR "controlled trial*")

S13 (MH "Controlled Before-After Studies" or MH "Historically Controlled Study" or MH "Interrupted Time Series Analysis" or MH "Nonrandomized Trials" or MH "[Pretest-Posttest Design](https://web.p.ebscohost.com/ehost/mesh/tree?term=Pretest-Posttest%20Design&sid=0819eb36-f54d-4784-84dc-de1c4ce7d525@redis&vid=21)+")

S14 TI ((clinical or controlled or (cross N1 over) or crossover or equivalent or pragmatic) N3 (studies or study or trial or trials)) or AB ((clinical or controlled or (cross N1 over) or crossover or equivalent or pragmatic) N3 (studies or study or trial or trials))

S15 TI ((interrupted N1 time) or (non N1 (randomised or randomized)) or nonequivalent or nonrandomised or nonrandomized) OR AB ((interrupted N1 time) or (non N1 (randomised or randomized)) or nonequivalent or nonrandomised or nonrandomized)

S16 TI ((one N1 group) and (((post or pre) N1 test) or pretest or posttest)) or AB ((one N1 group) and (((post or pre) N1 test) or pretest or posttest))

S17 TI (((pretest or (pre N5 (intervention or posttest or test))) and (posttest or (post N5 (intervention or test)))) or (pretest N5 posttest) or quasiexperiment* or (quasi N1 experiment*) or ((single N1 group) and (group N1 study)) or (uncontrolled N1 (studies or study))) or AB (((pretest or (pre N5 (intervention or posttest or test))) and (posttest or (post N5 (intervention or test)))) or (pretest N5 posttest) or quasiexperimental or (quasi N1 experimental) or ((single N1 group) and (group N1 study)) or (uncontrolled N1 (studies or study)))

S18 TI intervention

S19 S12 or S13 or S14 or S15 or S16 or S17 or S18

S20 S3 and S11 and S19

**Total: 1089**

Cochrane Central Register of Controlled Trials (Cochrane Library)

#1 MeSH descriptor: [Students, Nursing] this term only

#2 (nurs* near/2 (pupil* or student* or undergraduate* or baccalaureate* or graduate* or trainee* or educator*)):ti,ab,kw

#3 MeSH descriptor: [Computer Simulation] explode all trees

#4 MeSH descriptor: [Computer-Assisted Instruction] explode all trees

#5 MeSH descriptor: [Simulation Training] explode all trees

#6 (virtual* near/2 (realit* or realis* or train* or simulat* or educat* or instruct* or teach* or mentor* or learn* or patient* or environment* or hospital or ward or system* or setting* or world)):ti,ab,kw

#7 ((computer* or video* or patient*) near/2 simulat*):ti,ab,kw

#8 ((computer next assist* or computer next aid* or computer next based or computerized or video next assist* or video next aid*) near/2 (realit* or realis* or train* or simulat* or educat* or instruct* or self-instruct* or teach* or mentor* or learn*)):ti,ab,kw

#9 (simulat* near/2 (realit* or realis* or train* or educat* or instruct* or teach* or mentor* or learn*)):ti,ab,kw

#10 ((augmented or artificial) near/2 realit*):ti,ab,kw

#11 (VR or CAI or vSim or VRMagic or avatar* or Head-mounted Display* or HMD* or Cave Automatic Virtual Environment or CAVE or Oculus Rift or HTC Vive):ti,ab,kw

#12 (#1 or #2) and (#3 or #4 or #5 or #6 or #7 or #8 or #9 or #10 or #11)

**Total: 826 Trials matching "#12 - (#1 or #2) and (#3 or #4 or #5 or #6 or #7 or #8 or #9 or #10 or #11)"**

**Supporting Table 1.** GRADE Evidence Profile for Primary Outcomes.

| **Certainty assessment** | | | | | | | **No. of participants** | | **Effect** | **Certainty** | **Importance** |
| --- | --- | --- | --- | --- | --- | --- | --- | --- | --- | --- | --- |
| **No. of studies** | **Study design** | **Risk of bias** | **Inconsistency** | **Indirectness** | **Imprecision** | **Other considerations** | **IVR** | **Control** | **Absolute (95% CI)** |  |  |
| **knowledge acquisition** | | | | | | | | | | | |
| 11 | randomised trials | serious^a^ | serious^b^ | not serious | not serious | none | 457 | 413 | SMD **0.33** (95% CI 0.04 to 0.61) | ⨁⨁◯◯ Low | CRITICAL |
| **skill performance** | | | | | | | | | | | |
| 7 | randomised trials | serious^a^ | very serious^c^ | not serious | serious^d^ | none | 301 | 258 | SMD **0.49** (95% CI -0.24 to 1.22) | ⨁◯◯◯ Very Low | CRITICAL |

**CI:** confidence interval; **SMD:** standardized mean difference; **IVR:** immersive virtual reality simulation

a. Downgraded one level for risk of bias: majority of studies had some concerns or high risk in outcome measurement domain.

b. Downgraded one level for inconsistency: substantial heterogeneity (I² = 71.1%) partially explained by debriefing presence, but considerable heterogeneity remained within subgroups.

c. Downgraded two levels for inconsistency: very high heterogeneity (I² = 94.4%) with some reduction in certain subgroups but remaining substantial to very high overall.

d. Downgraded one level for imprecision: wide confidence interval (-0.24 to 1.22) crossing the threshold of no effect.

**Supporting Figure 1.** Risk of bias assessment for included studies: objective outcomes.

**Supporting Figure 2.** Risk of bias assessment for included studies: subjective outcomes.

**Supporting Figure 3.** Sensitivity analysis for knowledge acquisition outcomes


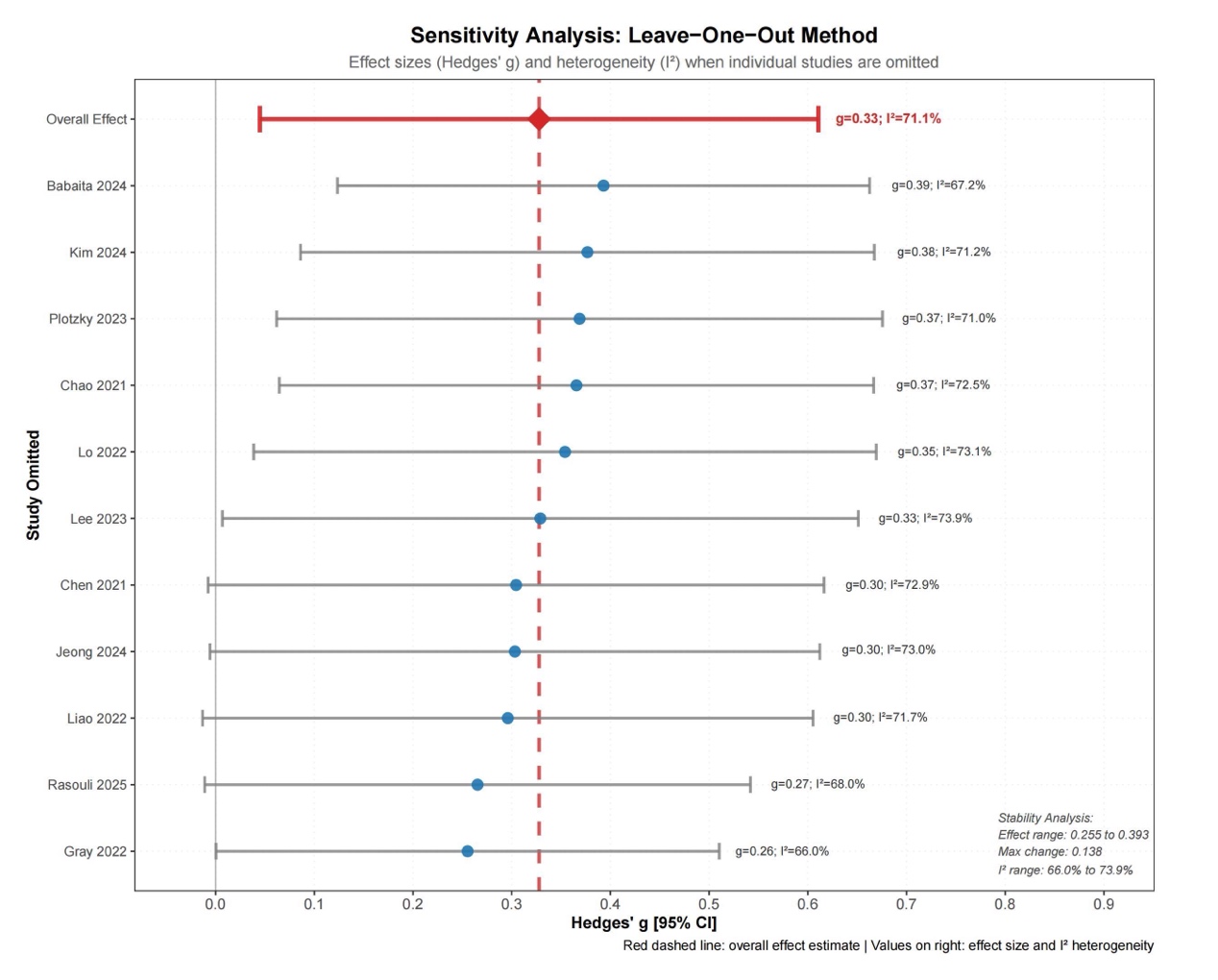


**Supporting Figure 4.** Funnel plot for knowledge acquisition outcomes

**Supporting Figure 5.** Subgroup analysis of knowledge acquisition outcomes by debriefing presence

**Supporting Figure 6.** Subgroup analysis of knowledge acquisition outcomes by follow-up timing

**Supporting Figure 7.** Sensitivity analysis for skill performance outcomes


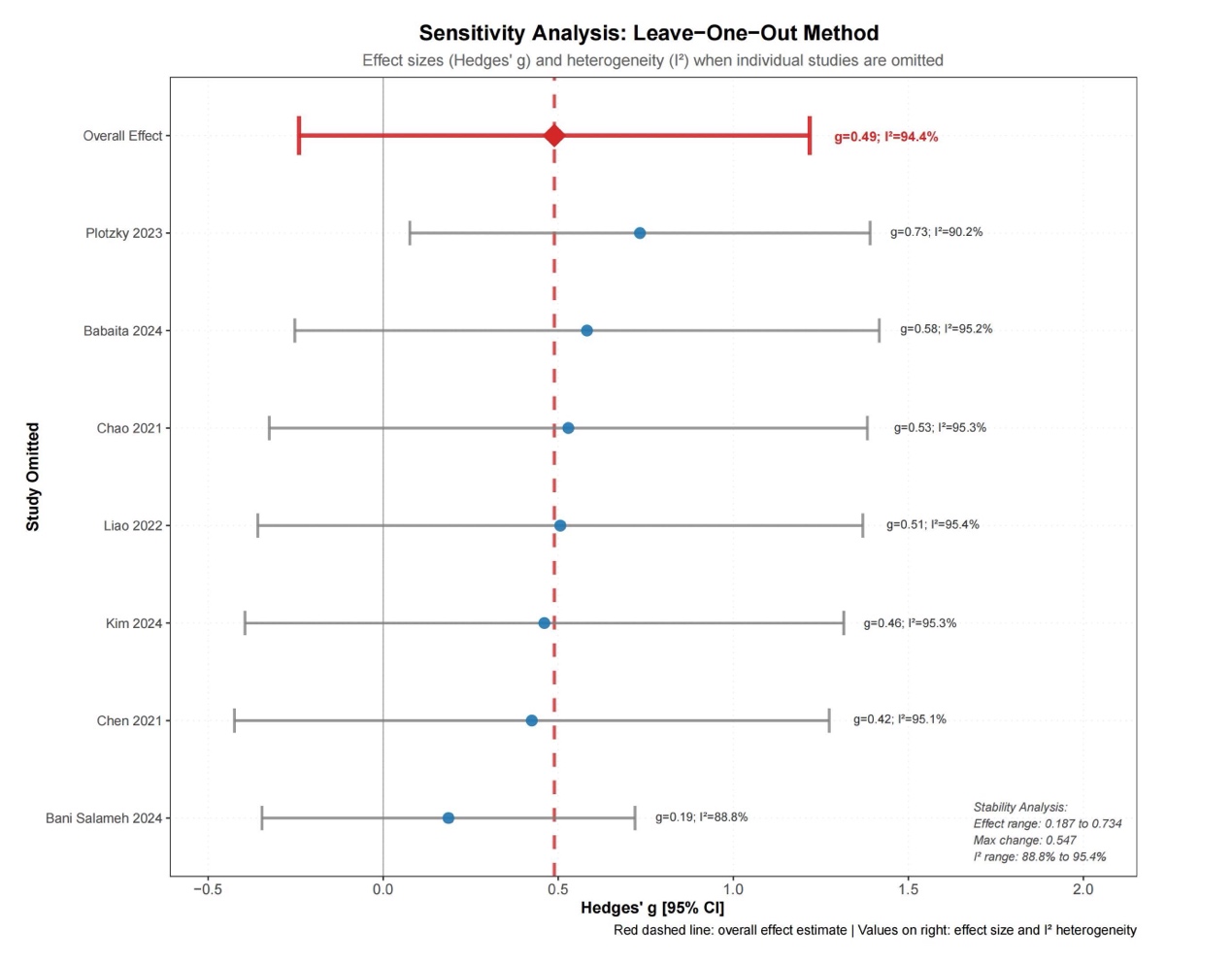


**Supporting Figure 8.** Funnel plot for skill performance outcomes

**Supporting Figure 9.** Subgroup analysis of skill performance outcomes by debriefing presence

**Supporting Figure 10.** Subgroup analysis of skill performance outcomes by follow-up timing

**Supporting Figure 11.** Forest plot for satisfaction outcomes


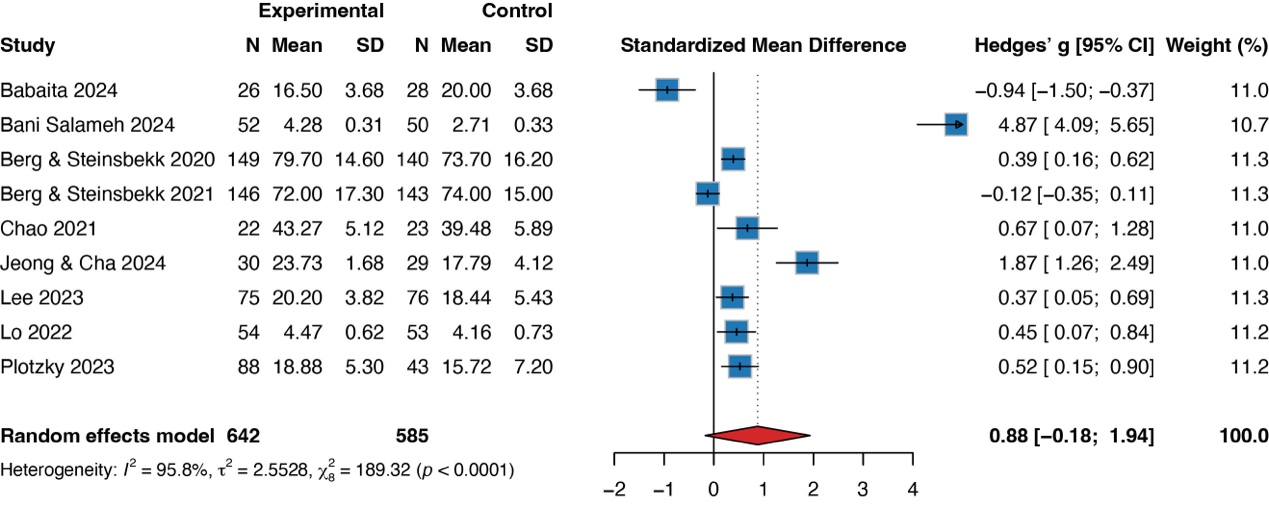


**Supporting Figure 12.** Forest plot for confidence outcomes


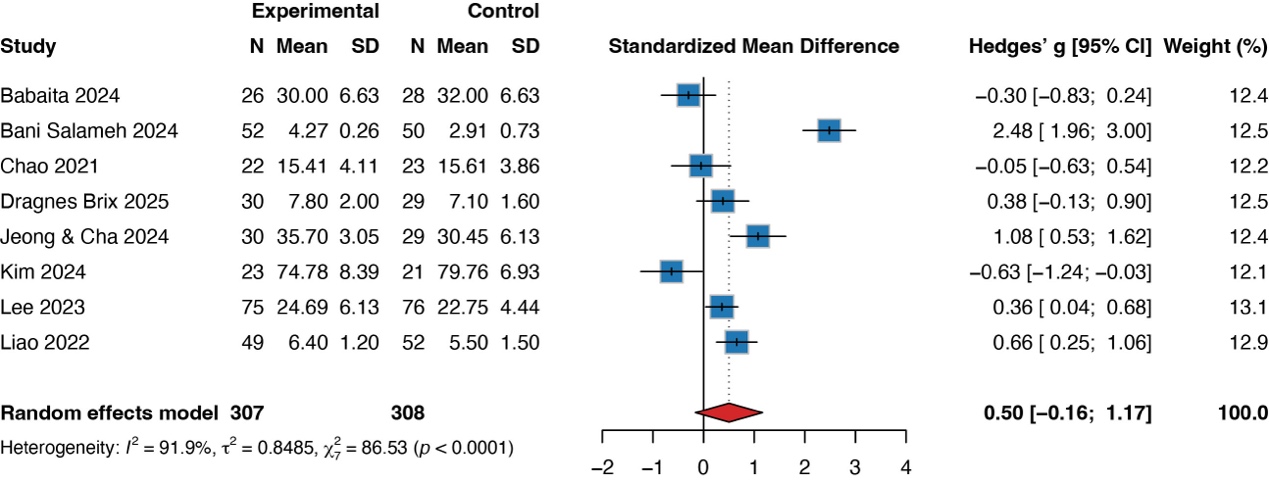


**Supporting Figure 13.** Forest plot for self-efficacy outcomes


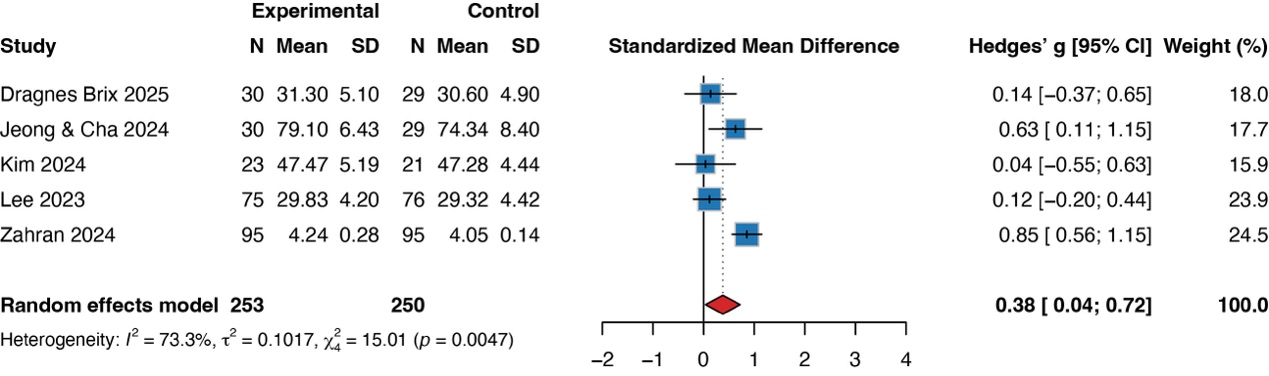


**Supporting Figure 14.** Sensitivity analysis for satisfaction outcomes


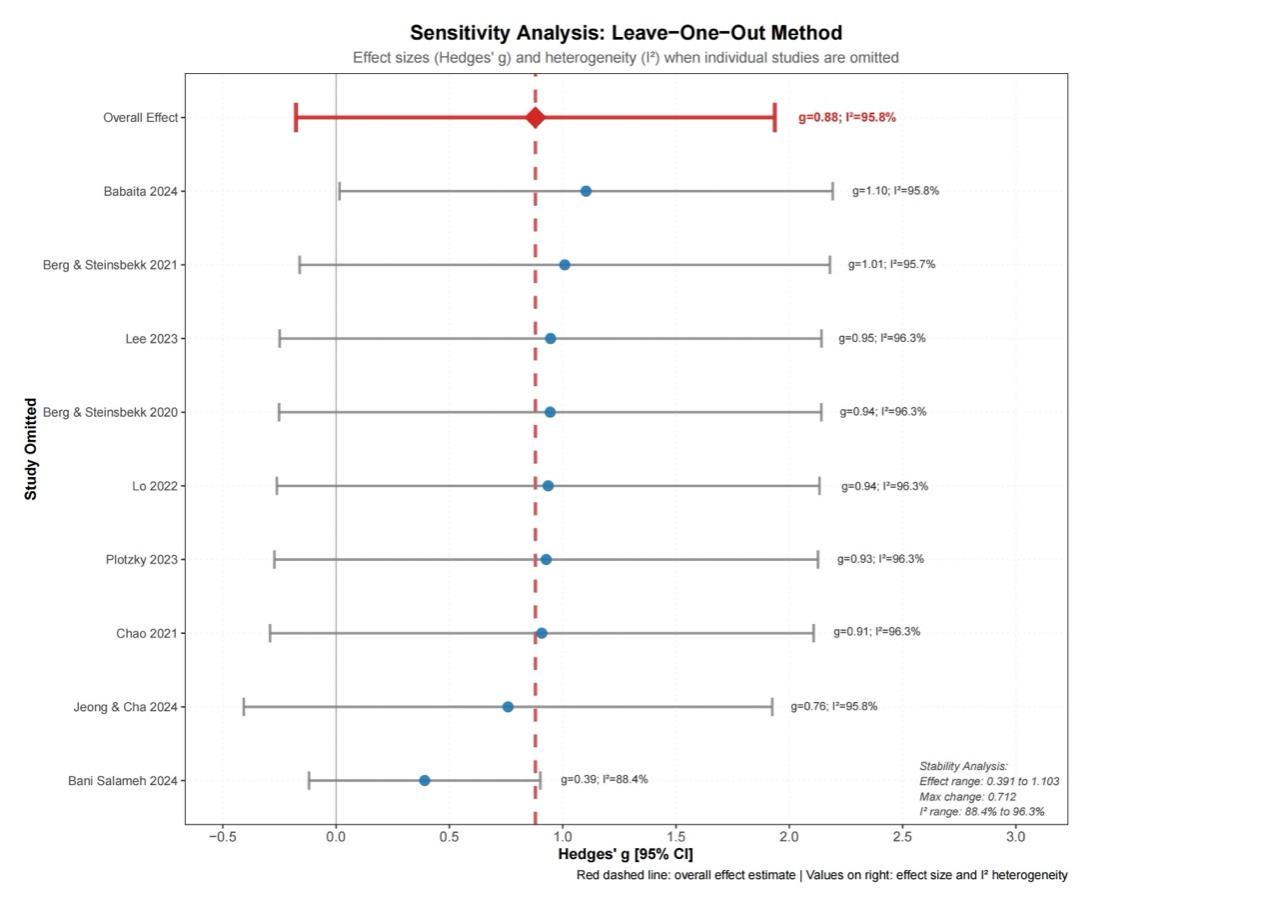


**Supporting Figure 15.** Sensitivity analysis for confidence outcomes


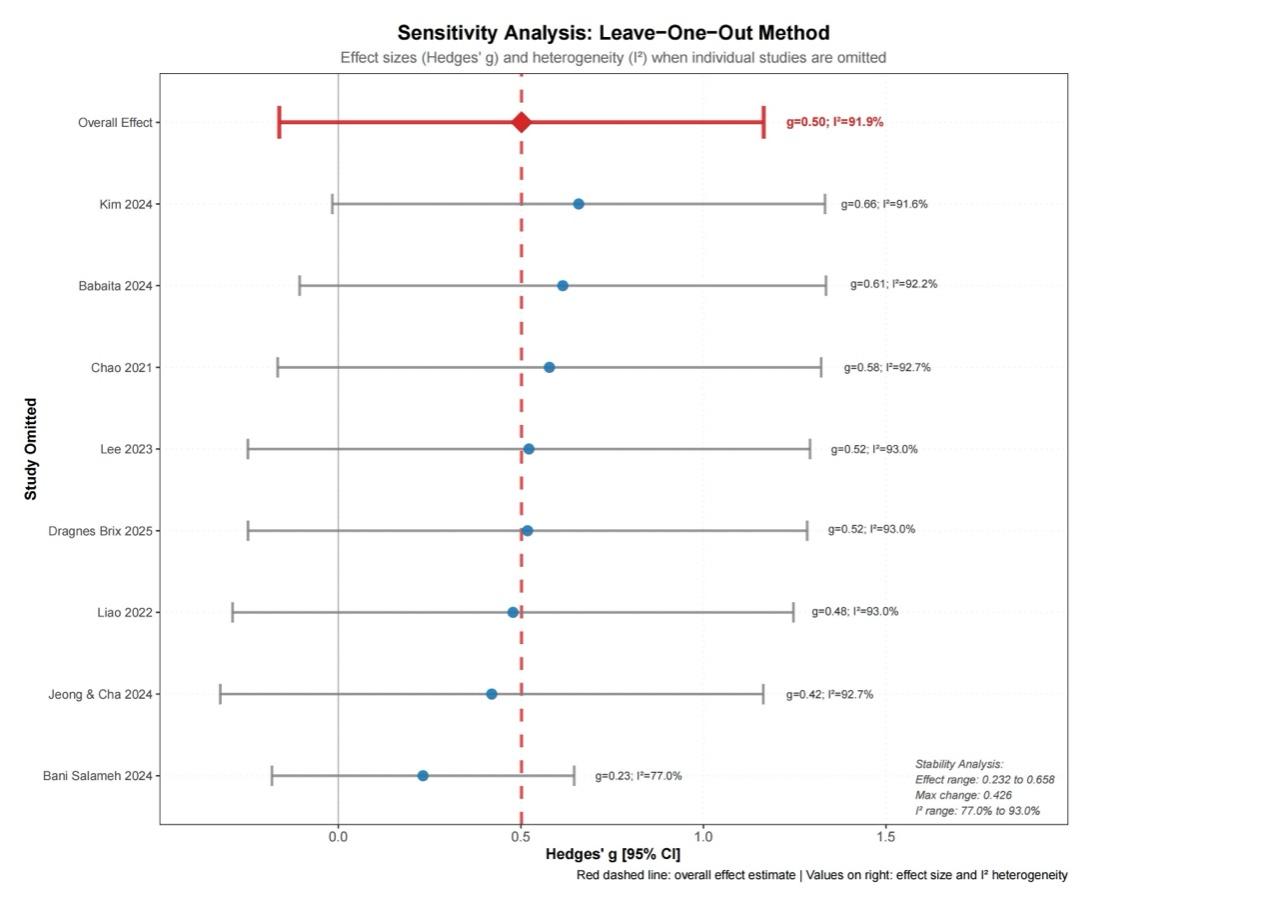


**Supporting Figure 16.** Sensitivity analysis for self-efficacy outcomes


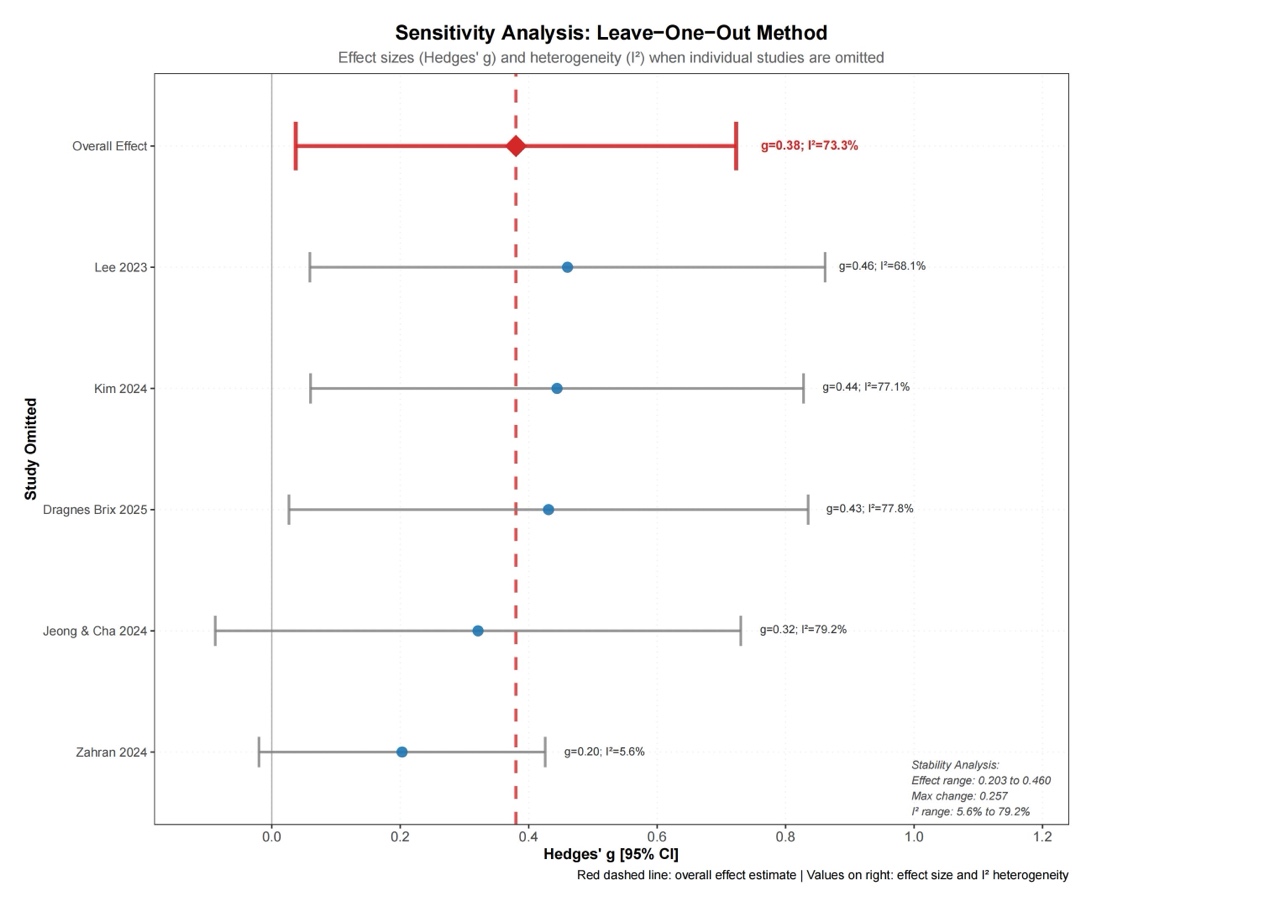


1. *Filters for identifying randomized trials in MEDLINE (#10) are available and have been adapted from https://training.cochrane.org/handbook/current/chapter-04#section-4-4-3* [↑](#footnote-ref-1)
2. *Filters for identifying non-randomized trials/quasi-experimental in MEDLINE (#11) are available and have been adapted from* [*https://hsls.libguides.com/Ovid-Medline-search-filters/experimental-studies#s-lg-box-32395726*](https://hsls.libguides.com/Ovid-Medline-search-filters/experimental-studies#s-lg-box-32395726) [↑](#footnote-ref-2)
3. *Filters for identifying randomized trials in Ovid Embase (#37) are available and have been adapted from https://sites.google.com/a/york.ac.uk/issg-search-filters-resource/home/rcts/embase-rct-filter* [↑](#footnote-ref-3)
